# Cell cycle inhibitors activate the hypoxia-induced DDX41/STING pathway to mediate antitumor immune response in liver cancer

**DOI:** 10.1172/jci.insight.170532

**Published:** 2024-11-22

**Authors:** Po Yee Wong, Cerise Yuen Ki Chan, Helen Do Gai Xue, Chi Ching Goh, Jacinth Wing Sum Cheu, Aki Pui Wah Tse, Misty Shuo Zhang, Yan Zhang, Carmen Chak Lui Wong

**Affiliations:** 1 Department of Pathology, School of Clinical Medicine, and; 2 State Key Laboratory of Liver Research, The University of Hong Kong, Hong Kong.; 3 Centre for Oncology and Immunology, Hong Kong Science Park, Hong Kong.; 4 Department of Clinical Oncology, The University of Hong Kong-Shenzhen Hospital, Shenzhen, China.; 5 Guangdong-Hong Kong Joint Laboratory for RNA Medicine, Sun Yat-Sen University, Guangzhou, China.

**Keywords:** Hepatology, Oncology, Cancer immunotherapy, Cell cycle, Liver cancer

## Abstract

Cell cycle inhibitors have a long history as cancer treatment. Here, we report that these inhibitors combated cancer partially via the stimulator of IFN genes (STING) signaling pathway. We demonstrated that paclitaxel (microtubule stabilizer), palbociclib (cyclin-dependent kinase 4/6 inhibitor), and AZD1152 and GSK1070916 (aurora kinase B inhibitors) have anticancer functions beyond arresting the cell cycle. They consistently caused cytosolic DNA accumulation and DNA damage, which inadvertently triggered the cytosolic DNA sensor DEAD-box helicase 41 (DDX41) and activated STING to secrete pro-inflammatory senescence-associated secretory phenotype factors (SASPs). Interestingly, we found that DDX41 was a transcriptional target of HIF. Hypoxia induced expression of DDX41 through HIF-1, making hypoxic hepatocellular carcinoma (HCC) cells more sensitive to the antimitotic agents in STING activation and SASP production. The SASPs triggered immune cell infiltration in tumors for cancer clearance. The treatment with cell cycle inhibitors, especially paclitaxel, extended survival by perturbing mouse HCC growth when used in combination with anti–PD-1. We observed a trend that paclitaxel suppressed Sting wild-type HCC more effectively than Sting-KO HCC, suggesting that STING might contribute to the antitumor effects of paclitaxel. Our study revealed the immune-mediated tumor-suppressing properties of cell cycle inhibitors and suggested combined treatment with immunotherapy as a potential therapeutic approach.

## Abstract 2

Cell cycle inhibitors induce cytosolic DNA accumulation which activates DDX41-STING pathway to secrete pro-inflammatory SASPs, thereby triggering innate immune response in hypoxic HCC.

## Graphical abstract

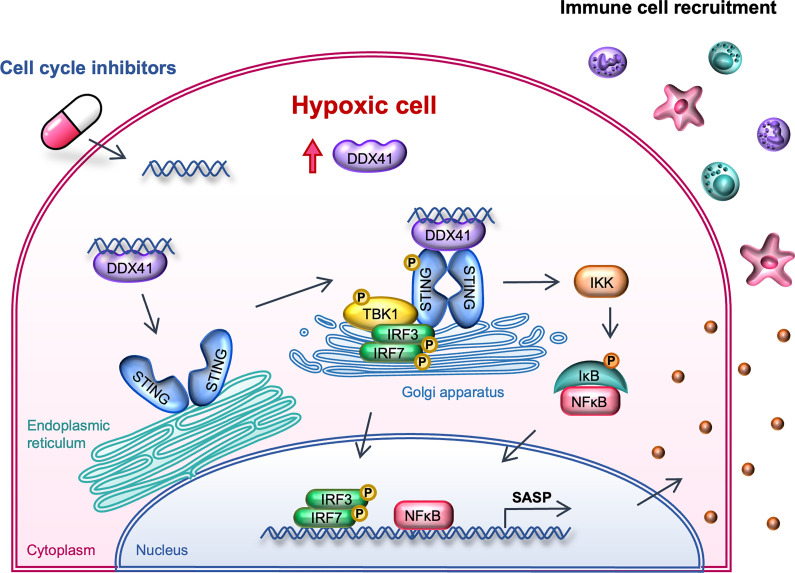

## Introduction

Hepatocellular carcinoma (HCC) is the sixth most common cancer in incidence and the third most common cause of cancer mortality worldwide (1). Cell cycle perturbation is often associated with carcinogenesis and malignant transformation and is a known feature of cancer (2, 3). Chemotherapy is one of the approaches that has been used to treat cancer for more than 50 years by inducing programmed cell death via cytotoxic or targeted manner (4). The classical cytotoxic therapies involve the use of paclitaxel (microtubule stabilizer) and palbociclib (cyclin-dependent kinase [CDK] 4 and 6 inhibitor), which have been approved by FDA for treating ovarian carcinoma, breast carcinoma, non–small cell lung carcinoma, AIDS-related Kaposi’s sarcoma (5), and breast cancer (6). Yet their effect toward HCC is still unclear as they are still in phase II clinical trials (NCT02423239 and NCT04175912 for paclitaxel) (NCT01356628 for palbociclib). Other cell cycle inhibitors, such as aurora kinase inhibitors (GSK1070916 and AZD1152), are now undergoing clinical trials and being investigated as potential therapies (7). However, some studies revealed that HCC built resistance toward paclitaxel (8, 9), and a phase II clinical study showed that paclitaxel did not give any survival advantage to patients with HCC (10). Besides, 2 out of 3 clinical trials of AZD1152 on advanced solid tumor were terminated, as the efficacy of AZD1152 monotherapy was not sufficient to support the continuity of research (NCT00497679, NCT00338182, and NCT00497731), suggesting the cell cycle inhibitors in general might not be beneficial to HCC; monotherapy and other approaches, for example combination therapy with other tyrosine kinase inhibitors (TKIs) or immune checkpoint inhibitors (ICIs), might be needed for enhancing the effect of cell cycle inhibitors.

Since HCC is refractory to chemotherapies, systemic therapies are adopted for advanced HCC (11). The FDA-approved first-line therapies, including the TKIs sorafenib and lenvatinib, benefit patients with advanced HCC with extended overall survival (OS) of 3 months only, while the second-line therapies increase OS to 1.6–2.8 months in tumor-progressing sorafenib-treated patients (12, 13).

ICIs in the past few years showed better efficacy over the traditional TKI regimen, and the FDA has recently approved a combination of ICI atezolizumab and anti-VEGF antibody bevacizumab as first-line therapy with better median OS compared with sorafenib (13–15). However, the results of other ICI monotherapy, for instance anti–programmed cell death 1 (anti–PD-1), were not so promising and did not improve the survival endpoint due to low response rate (12, 16). Hence, in this study, we aimed to figure out new combination therapies to boost the efficacy of current medication.

Stimulator of IFN genes (STING) is an adaptor protein involved in the antiviral signaling pathway. It triggers the expression of type 1 IFN to initiate innate immune response (17–21). Our previous study showed that polo-like kinase 4 inhibitor caused micronuclei-induced cytosolic DNA, which triggered the expression of senescence-associated secretory phenotype factors (SASPs) via the DEAD-box helicase 41 (DDX41)/STING/TANK-binding kinase 1 (TBK1)/interferon regulatory factor 3/7 (IRF3/7)/NF-κB pathway to activate immune response (22). Here, we explore whether this mechanism can be generally applied to other well-known but mechanistically distinct inhibitors targeting different cell cycle phases, through apoptosis-mediated DNA release, which would further activate the DDX41/STING pathway. This response was even more dramatic under the hypoxic condition, which was usually found in solid tumors. Our study opened the avenue of combining cell cycle inhibitors with ICIs to halt cell proliferation and concurrently unleash the full potential of immune cells to eradicate cancer.

## Results

### Overexpression of key cell cycle regulators in HCC is correlated to poor OS

**Figure 1:**
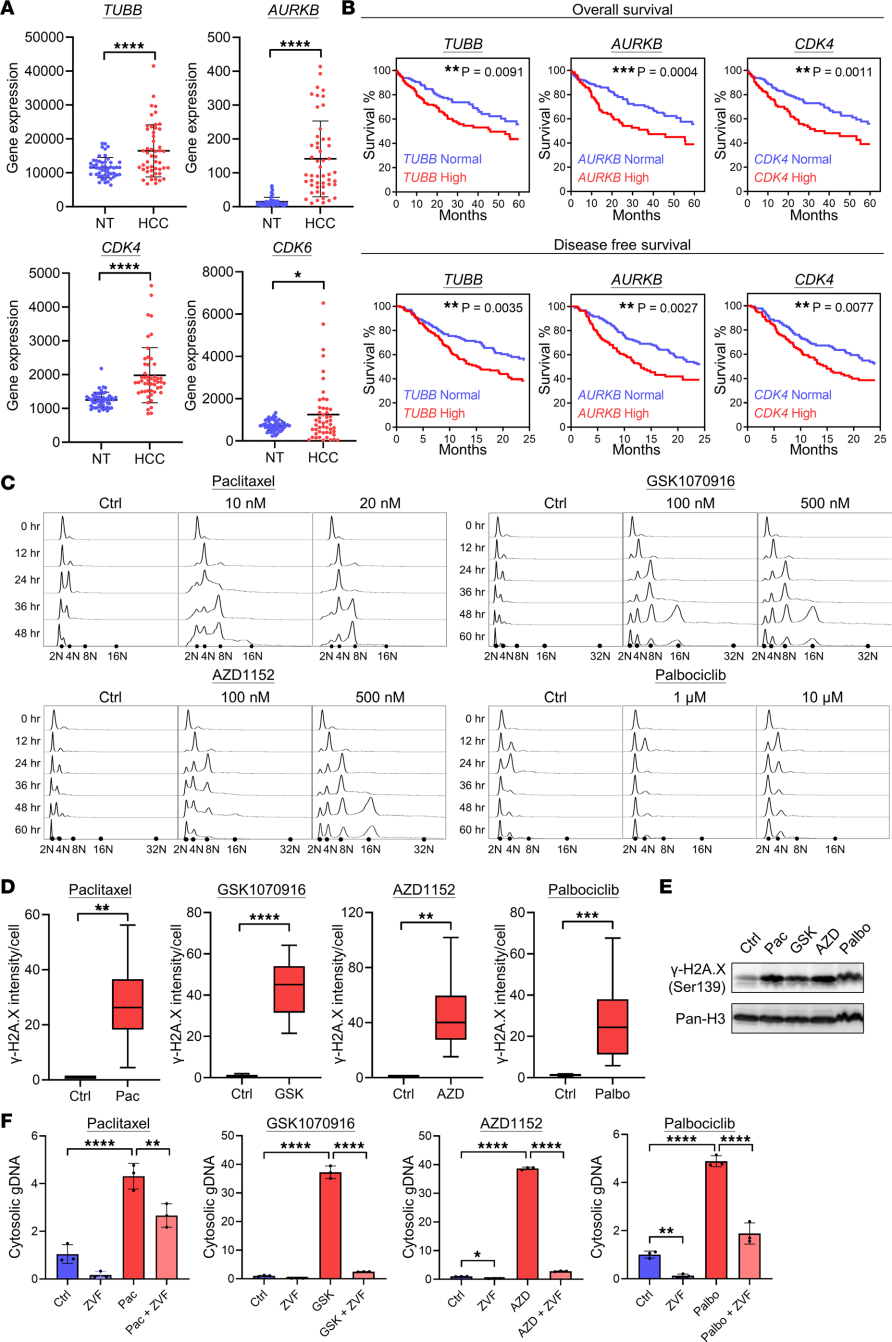
Cell cycle inhibitors lead to genome instability, DNA damage, and cytosolic DNA accumulation in cell cycle regulator–high HCC cells. (**A**) TCGA data showing the upregulated gene expression level (RNA-Seq by expectation maximization normalized count) of tubulin (*TUBB*), aurora kinase B (*AURKB*), *CDK4*, and *CDK6* in HCC tissues compared to corresponding nontumorous liver tissues (NT) in patients with HCC (*n* = 50/group). Scatter dot plot: mean with SD. Student’s *t* test. (**B**) TCGA data showing the correlation between HCC patients with normal or high expression level of *TUBB*, *AURKB*, and *CDK4* and the percentage of survival in OS and disease-free survival model (*Z* > 0). (*n* > 300/plot.) Log-rank (Mantel-Cox) test. (**C**) MHCC97L cells were synchronized using double thymidine method. The cell cycle inhibitors with the indicated concentration were applied to the cells. The DNA content was detected using propidium iodide (PI). Number of cells analyzed in each treatment (*N*) ≥ 10,000. (**D** and **E**) MHCC97L cells were treated with cell cycle inhibitors for 48 hours. (**D**) DNA damage was detected using γ-H2A.X in immunofluorescence (IF) staining. Number of cells analyzed in each treatment (*N*) ≥ 85. Box and whiskers: min to max. Student’s *t* test. (**E**) After histone extraction, Western blot was used to show the expression level of γ-H2A.X. (**F**) MHCC97L cells were treated with cell cycle inhibitors alone or ZVF alone or cotreated with both cell cycle inhibitors and ZVF for 72 hours. The cells were extracted for cytosolic DNA and total DNA. The gDNA was detected using qPCR. The cytosolic gDNA was determined by normalizing the relative expression of gDNA in the cytoplasmic portion to that in the total portion (*n* = 3/group). Scatter dot plot: mean with SD. (**A**–**D**) Student’s *t* test. (**F**) One-way ANOVA with Bonferroni’s correction. *P < 0.05, **P < 0.01, ***P < 0.001, ****P < 0.0001.

The Cancer Genome Atlas (TCGA) data showed that the gene expression levels of tubulin (*TUBB*), aurora kinase B (*AURKB*), *CDK4*, and *CDK6* were all upregulated in HCC tissues as compared with corresponding nontumor (NT) liver tissues (Figure 1A). The expression levels of these cell cycle proteins in HCC were negatively correlated to overall and disease-free survival (Figure 1B). This showed the importance of cell cycle proteins in HCC development and patient OS by promoting cell growth and proliferation. Accordingly, impeding the cell cycle by targeting these proteins using inhibitors may block and cease HCC development.

### Cell cycle inhibitors lead to genome instability, DNA damage, and cytosolic DNA accumulation in HCC cells

#### Cell cycle perturbation and genome instability.

Cell cycle profiles were determined to monitor the effect of different cell cycle inhibitors. Paclitaxel, GSK1070916, AZD1152, and palbociclib were used to interrupt the cell cycle progression of double thymidine–synchronized HCC cell lines MHCC97L and CLC4. Their cell cycle profiles were monitored by flow cytometry (FC). The DNA content of cells shifted from 2N to 4N after 12 hours in MHCC97L and 24 hours in CLC4. Furthermore, a broad peak ranging from 2N to 8N was observed at 36 hours in MHCC97L and 48 hours in CLC4 after treatment of paclitaxel (Figure 1C and Supplemental Figure 1; supplemental material available online with this article; https://doi.org/10.1172/jci.insight.170532DS1). Similar results were obtained with AZD1152 and GSK1070916 where 8N and 16N peaks appeared in both MHC97L and CLC4 after 48 hours and 72 hours, respectively. Palbociclib showed a different profile where the peak shifted back to 2N after 24 hours and no 8N or 16N peak was observed. The presence of aneuploidy, indicated by broad peaks ranging between 2N and 4N and between 4N and 8N peaks and polyploidy, indicated by 8N and 16N peaks, suggested most but not all antimitotic agents caused accumulation of genome instability in cells.

#### Increase of ROS level.

The cytotoxic property of the cell cycle inhibitors might also boost their genotoxicity. The inhibitors might introduce oxidative stress to the cells by generating ROS (23–25). We examined whether the inhibitors add oxidative stress to the cells by detecting the cellular ROS level using CM-H_2_DCFDA, a fluorogenic dye that becomes highly fluorescent after being oxidized by ROS in cells. The cellular ROS level increased after treating the cells with the inhibitors, suggesting the inhibitors were cytotoxic and generated oxidative stress in the HCC cells (Supplemental Figure 2).

#### DNA damage.

The improper cell division and the accumulation of extensive DNA might introduce stress in chromosomes and cause DNA damage and accumulation of DNA in cytoplasm. The buildup of cellular ROS could also cause harm to DNA by intensifying the replication stress, generating DNA double-strand breaks, hampering DNA repair, oxidizing the nucleoside bases, and promoting oxidative DNA damage (26, 27). Therefore, we examined if DNA damage occurred in cells using gamma histone 2A.X (γ-H2A.X) as the DNA damage marker. Both immunofluorescence (IF) data (Figure 1D and Supplemental Figure 3) and Western blot (Figure 1E) showed an increase in γ-H2A.X level, in cell cycle inhibitor–treated cells, verifying the inhibitors induced DNA damage in cells.

#### Apoptosis.

Genome integrity is indispensable for the proliferating of life. Chromosome mis-segregation induced aneuploidy and polyploidy, promoted chromosome instability, affected the maintenance of genome integrity, and eventually led to programmed cell death via apoptosis (28). Besides, other cellular stress, such as DNA damage, hypoxia, and oxidative stress that are brought by increased ROS would also provoke apoptosis. Hence, we detected cell cycle inhibitor–induced apoptosis by using annexin V and propidium iodide (PI) assay, which discriminates live cells from early and late apoptotic cells. We found that all the inhibitors generated apoptotic cell death in MHCC97L cells (Supplemental Figure 4). Z-VAD-Fmk (ZVF) is a pan-caspase inhibitor that inhibits apoptosis. The apoptosis could be rescued by ZVF, further verifying that the cell cycle inhibitors induced cell death through apoptosis.

#### Cytosolic DNA accumulation.

We compared the relative amount of genomic DNA (gDNA) in cytoplasm to see if cell cycle inhibitors caused DNA accumulation in cytoplasm. The cytosolic gDNA isolated from the cells was measured using quantitative PCR (qPCR) and normalized to total gDNA. The qPCR data showed the gDNA content detected in cytoplasm increased significantly after drug treatment (Figure 1F). Our data demonstrated that only paclitaxel and aurora kinase B inhibitors induced aneuploidy and polyploidy (Figure 1C), and all inhibitors caused accumulation of cytosolic gDNA.

Since apoptosis involves DNA fragmentation by caspase-activated DNase, the fragments may leak from the nucleus and contribute to accumulation of cytosolic DNA (29–32). In order to verify this hypothesis, we suppressed apoptosis by using ZVF to see if the accumulation of gDNA in cytoplasm was abolished. We found that ZVF substantially reduced the cytosolic gDNA level in the presence of cell cycle inhibitors, verifying that apoptosis was essential for the buildup of cytosolic gDNA and indicating it was the major source of cytosolic gDNA (Figure 1F).

### Cell cycle inhibitors induce cellular senescence and SASP secretion

**Figure 2:**
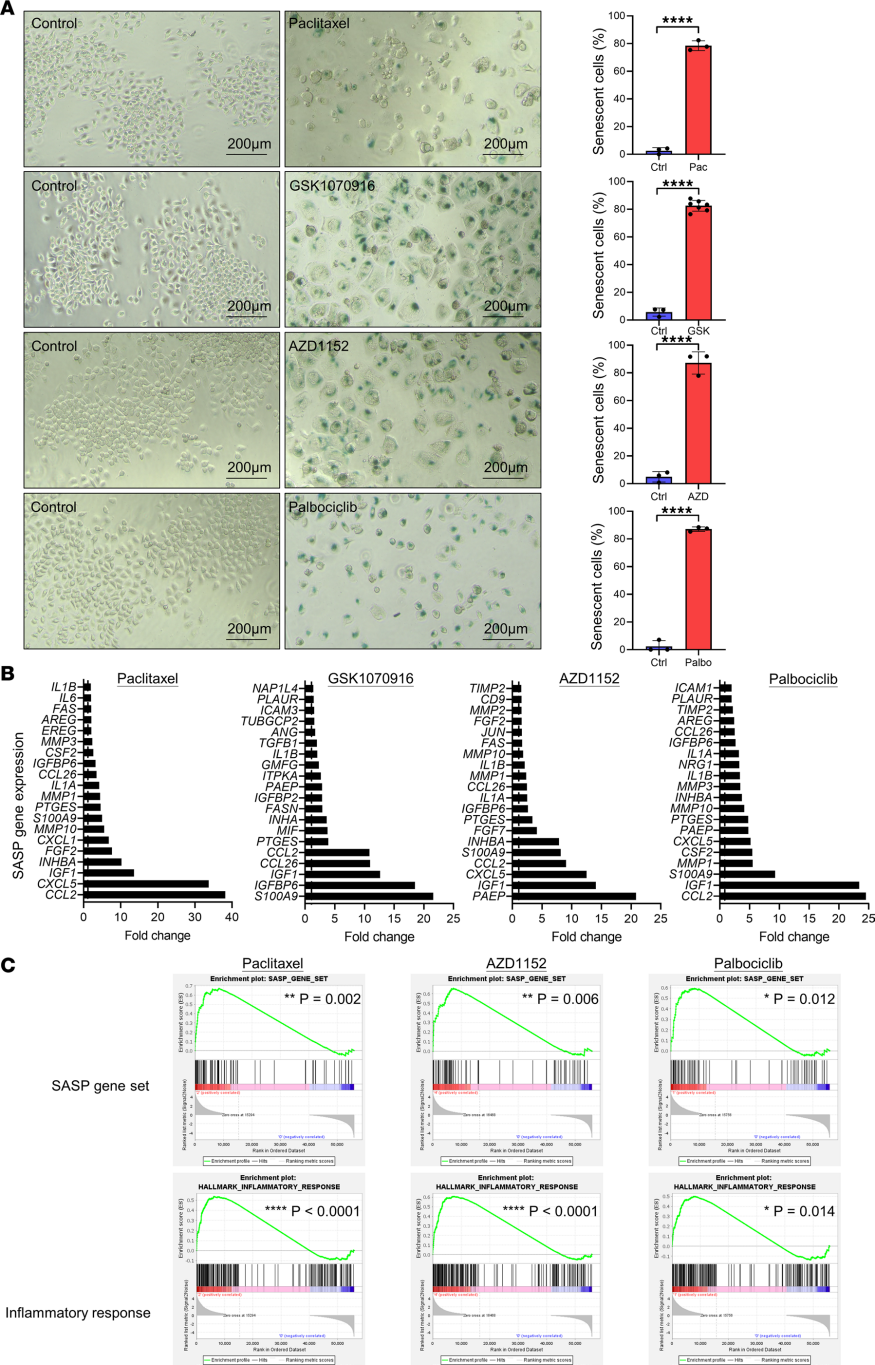
Cell cycle inhibitors induce cellular senescence and SASP secretion. MHCC97L cells were treated with cell cycle inhibitors for 96 hours. (**A**) Senescent cells were detected using β-gal staining, and cell images were captured and analyzed using an inverted microscope. Transparent, nonsenescence; blue, senescence. Number of cells analyzed in each treatment (*N*) ≥ 200. Scale: 200 μm. Scatter dot plot: mean with SD. (**B** and **C**) RNA was extracted and prepared for RNA sequencing. Number of cells analyzed in each treatment (*N*) ≥ 1 × 10^5^. (**B**) The fold-change of the gene mRNA expression level was analyzed. The top 20 SASP genes with highest fold increase in expression level were extracted and visualized on the graph. (**C**) RNA-sequencing data were used to analyze the enrichment of inflammatory response and SASP gene set using gene set enrichment analysis (GSEA). (**A**–**C**) Student’s *t* test. *P < 0.05, **P < 0.01, ****P < 0.0001.

Senescence occurs when cells enter permanent growth arrest and become nonproliferative. Since it can be induced by stress, for example oxidative stress and DNA damage (33), we examined if cell cycle inhibitors induce cellular senescence by detecting senescence-associated β-galactosidase (SA-β-Gal) activity in cells, which is indicated by blue color (Figure 2A). The cells turned blue after treatment with the cell cycle inhibitors, and the morphology of cells became flat and enlarged, showing that the cells underwent senescence in the presence of all inhibitors. Since senescent cells secrete SASPs, we detected the mRNA level of SASPs in cells under different inhibitors using RNA-Seq (Figure 2B). Only the top 20 SASP genes with highest fold-change in mRNA level were shown in the plot, and the data illustrated all inhibitors promoted expression of different SASPs. Among the 4 inhibitors, the expression of CCL2 increased the most, and thus in the later experiments, CCL2 was used as the representative of SASPs. Gene set enrichment analysis (GSEA) also showed the expression of the SASP gene set was upregulated under treatment with paclitaxel, AZD1152, and palbociclib. Since SASP consists of cytokines and chemokines that take part in inflammatory and immune response, we analyzed the gene sets of inflammatory response, cytokine, TNF-α signaling via NFκB, and IL6-JAK-STAT3 signaling and found that they were also enriched in the presence of cell cycle inhibitors (Figure 2C and Supplemental Figure 5). In addition, these antimitotic agents also upregulated *CCL2* mRNA level in CLC4 cells (Supplemental Figure 6).

### Cell cycle inhibitors induce SASP secretion via the DDX41/STING pathway

**Figure 3:**
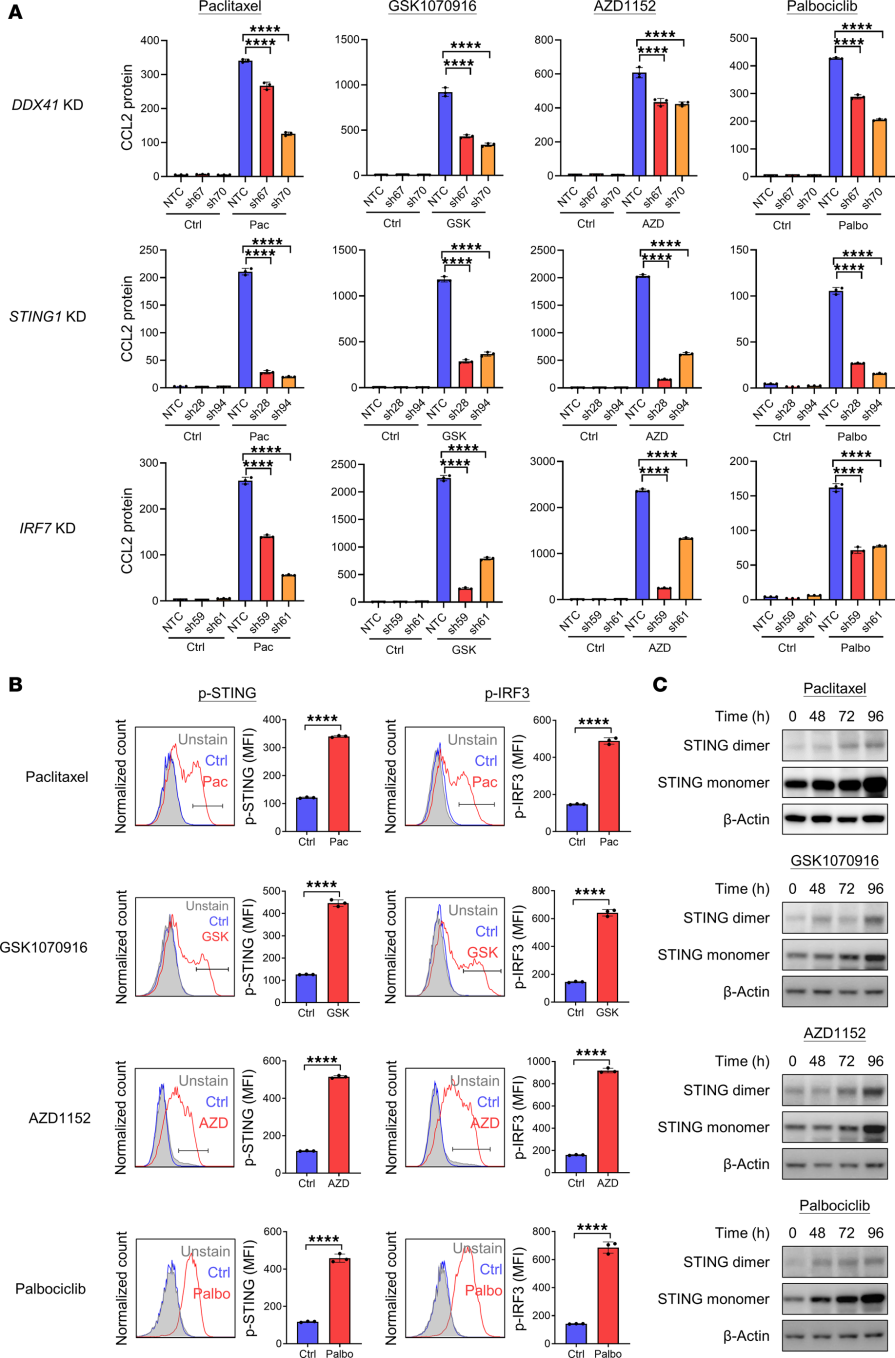
Cell cycle inhibitors induce SASP secretion via the DDX41/STING pathway. (**A**) MHCC97L stable clones of *DDX41*, *STING1*, and *IRF7* KD were treated with cell cycle inhibitors for 96 hours and were released in drug-free medium with 2% FBS for 24 hours. The medium was collected and the cell numbers were counted. The concentration of CCL2 that secreted into the medium was determined using ELISA. The CCL2 protein concentration was normalized to the cell number (pg/mL/100,000 cell). (**B**) MHCC97L cells were treated with 10 nM paclitaxel, 100 nM GSK1070916, 100 nM AZD1152, or 10 μM palbociclib for 96 hours. Phosphorylated STING (p-STING) and phosphorylated IRF3 (p-IRF3) were detected using specific antibodies and analyzed using FC (*n* = 3/group). (**C**) MHCC97L cells treated with cell cycle inhibitors were collected at indicated hours, and the cell lysate was extracted. Western blot was used to show the protein expression level of STING monomer and dimer, and β-actin was used as a housekeeping protein. Scatter dot plot: mean with SD. (**A**) One-way ANOVA with Bonferroni’s correction. (**B**) Student’s *t* test. ****P < 0.0001.

It has been reported that the DNA sensor DDX41 was able to sense cytosolic DNA and activate the STING/TBK1/IRF3/7 pathway to induce SASP expression (22). Upon activation by DDX41, STING dimerized in a *V* shape and translocated from the ER to Golgi apparatus to recruit TBK1. TBK1 phosphorylated STING and further phosphorylated and recruited IRF3/7 and NF-κB to the nucleus for transcription of IFN-β, IL-6, and TNF (18–21). Hence, we determined whether this pathway was used in the case of cell cycle inhibitor treatment by knocking down the prime components in the pathway. Both qPCR and ELISA data illustrated that knockdown (KD) of *DDX41*, *STING1*, *IRF7*, and *RelA* (transcription factor p65, subunit of NF-κB) abrogated expression of CCL2 (Figure 3A and Supplemental Figure 7, A and B). FC data demonstrated the phosphorylation of STING and IRF3 after cell cycle inhibitor treatment in both MHCC97L and CLC4 (Figure 3B and Supplemental Figure 8), and the Western blot data showed that cell cycle inhibitors caused STING dimerization in a time-dependent manner (Figure 3C). Our data suggested that cell cycle inhibitors caused accumulation of DNA in cytoplasm, which triggered DDX41 to activate the STING/TBK1/IRF3/7 pathway to initiate SASP secretion.

### Hypoxia further exaggerates the effect of cell cycle inhibitors on SASP secretion

**Figure 4:**
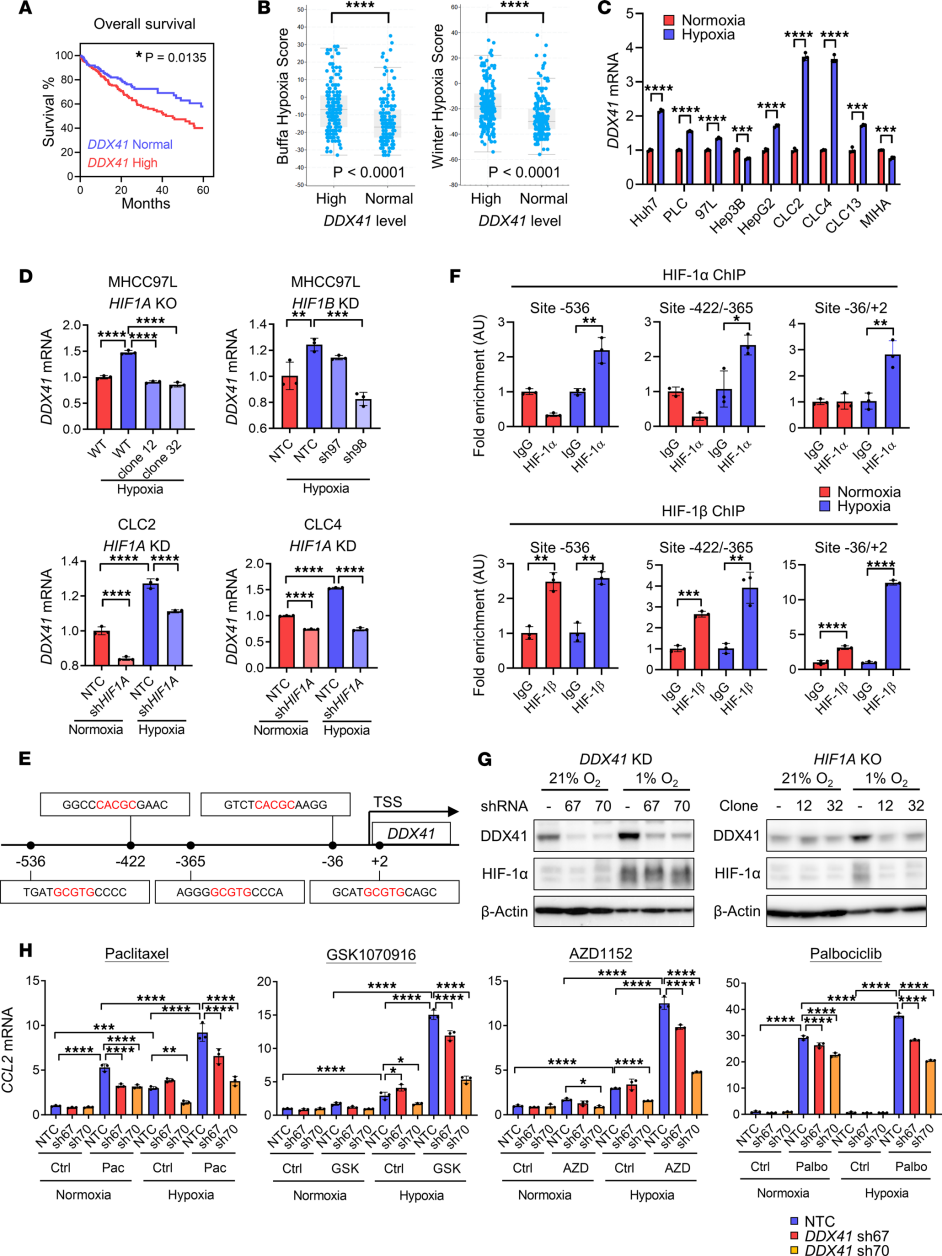
Hypoxia further exaggerates the effect of cell cycle inhibitors on SASP secretion via upregulating DDX41. (**A**) TCGA data showing the correlation of *DDX41* expression levels and overall survival in patients with HCC (*n* = 165 in *DDX41*-normal group; *n* = 200 in *DDX41*-high group). The high *DDX41* expression level was defined as *DDX41* mRNA expression higher than the mean value (*Z* > 0). Log-rank (Mantel-Cox) test. (**B**) TCGA data showing the Buffa hypoxia and Winter hypoxia score with *DDX41* expression level in patients with HCC. Wilcoxon test. (**C**) The HCC cell lines and immortalized hepatocytes MIHA were exposed to normoxia (21% O_2_) or hypoxia (1% O_2_) for 24 hours. The mRNA expression of *DDX41* was determined using reverse transcription quantitative PCR (RT-qPCR) and normalized to housekeeping gene *18S*. (*n* = 3/group.) (**D**) HIF1 subunit–knockdown and –knockout cell lines were exposed to normoxia or hypoxia for 24 hours. *DDX41* mRNA expression in hypoxia was normalized to that in wild-type (WT) cells or NTC cells in normoxia (*n* = 3/group). (**E**) Diagram showing 5 putative HRE sites in the promotor region of *DDX41* where the transcription start site (TSS) is defined as 0. Red: HRE site. (**F**) MHCC97L cells were exposed to normoxia with 21% O_2_ or hypoxia with 1% O_2_ for 24 hours. The binding of HIF-1α or HIF-1β to the HRE sites of *DDX41* was detected in chromatin immunoprecipitation (ChIP) assay. The enrichment of HIF-1α or HIF-1β was analyzed using qPCR with primers targeting different HRE sites in *DDX41* promoter. (*n* = 3/group.) (**G**) Western blot was used to show the expression level of DDX41 and HIF-1α. (**H**) *DDX41*-KD MHCC97L cells were treated with paclitaxel, GSK1070916, or AZD1152 at the same time when exposed to normoxia with 21% O_2_ or hypoxia with 1% O_2_ for 48 hours. The *CCL2* mRNA expression level was normalized to that in control treatment in NTC cells in normoxia (*n* = 3/group). Scatter dot plot: mean with SD. (**A**–**C** and **F**) Student’s *t* test. (**D** and **H**) One-way ANOVA with Bonferroni’s correction. *P < 0.05, **P < 0.01, ***P < 0.001, ****P < 0.0001.

TCGA data revealed that upregulation of *DDX41* was correlated with poor OS in HCC (Figure 4A). The high *DDX41* level was also associated with higher Buffa and Winter hypoxia scores (Figure 4B), suggesting DDX41 may be hypoxia related. Hypoxia is defined as the reduction of oxygen availability or oxygen partial pressure that hampers the tissue or cell function, which can be caused by cancer (34). Hence, we checked the *DDX41* mRNA level in different HCC cell lines under hypoxia to see if *DDX41* was induced. We found that *DDX41* was upregulated under hypoxia in most of the HCC cell lines but not MIHA, the immortalized human hepatocytes (Figure 4C). Since most of the hypoxic events are regulated by HIF-1, which mainly comprises HIF-1α and HIF-1β, we verified if the hypoxia-induced expression of *DDX41* was regulated by HIF. Knockout (KO) of *HIF1A* and KD of *HIF1B* in MHCC97L cells suppressed the upregulation of *DDX41* under hypoxia (Figure 4D). The expression of DDX41 was upregulated under hypoxia in nontarget control (NTC) and KD groups when compared with normoxic condition in CLC2. However, KD of *HIF1A* in CLC2 and CLC4 HCC cell lines also impaired the expression of *DDX41* in hypoxia compared with NTC control (Figure 4D). Then we asked if *DDX41* is a direct target of HIF. Five putative hypoxia-response elements (HREs) were found in the promoter region of *DDX41* and were designed to be covered by 3 primer pairs (Figure 4E). Chromatin immunoprecipitation (ChIP) assay revealed the enrichment of HIF-1α binding to HRE of *DDX41* only under hypoxia while the binding of HIF-1β to *DDX41* was enriched in both normoxic and hypoxic conditions (Figure 4F). Western blot verified the augmented DDX41 protein expression under hypoxia where the expression was reduced when *DDX41* was knocked down or when *HIF1A* was knocked out (Figure 4G). These results showed the expression of DDX41 was regulated directly under hypoxia via HIF-1α and HIF-1β. We asked whether cell cycle inhibitors could elicit cytosolic DNA sensing to activate transcriptions of SASPs in hypoxic HCC cells, as DDX41 is directly regulated by HIF-1. The qPCR data evidenced that the expression of *CCL2* in cell cycle inhibitor–treated cells was elevated significantly under hypoxia after paclitaxel, AZD1152, GSK1070916, and palbociclib treatments. The increment was repressed when *DDX41* was knocked down (Figure 4H). Our data demonstrated that the effect of cell cycle inhibitors on SASP expression was boosted significantly in hypoxic tumor due to the upsurge of HIF-regulated DDX41.

### Cell cycle inhibitors induce immune surveillance in HCC tumors and promote survival in combination with ICIs

**Figure 5:**
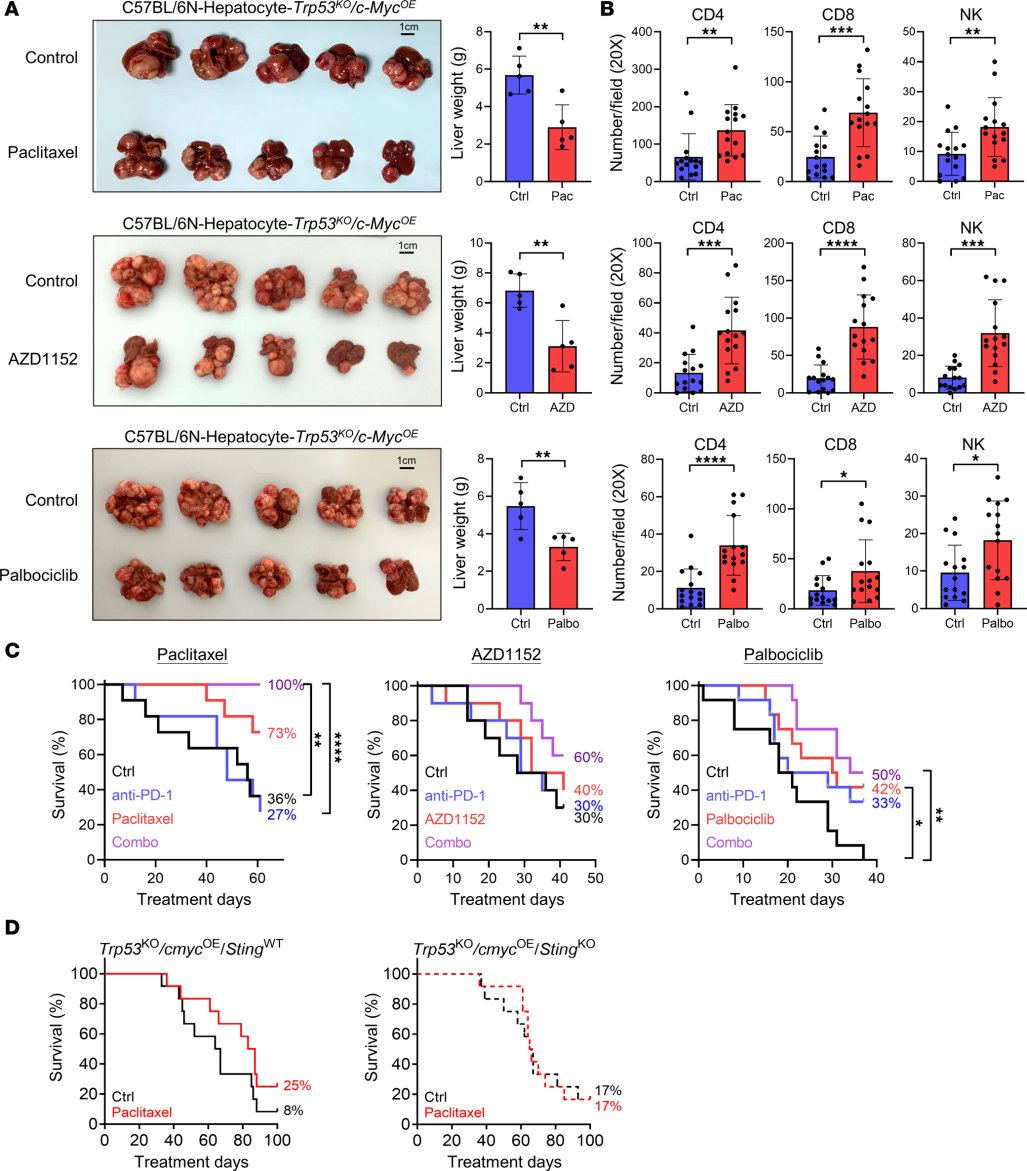
Cell cycle inhibitors induce immune surveillance in HCC tumors and promote survival in combination with an ICI. (**A** and **B**) Cell cycle inhibitors were administrated to *Trp53^KO^ c-Myc^OE^* HCC tumor–bearing C57BL/6N mice 3 weeks after HDTVi. The tumors were harvested 28 days after paclitaxel treatment, 20 days after AZD1152 treatment, or 16 days after palbociclib treatment (*n* = 5 mice per group). (**A**) Images showing the size and morphology of HCC tumors in control and cell cycle inhibitor–treated mice. Scale: 1 cm. The liver weight was measured. (**B**) The tumors were fixed and prepared for paraffin-embedded slices (plots were analyzed with 3 sections from each mouse, in total 15 sections from 5 mice). CD4^+^ T cells, CD8^+^ T cells, and NK cells were detected using mouse CD4, CD8α, and KLrb1c/CD161c antibodies in IHC staining. The numbers of CD4^+^ T cells, CD8^+^ T cells, and NK cells were quantified per field in paraffin-embedded slices of control and cell cycle inhibitor–treated mice. Scatter dot plot: mean with SD. Student’s *t* test. (**C**) Vehicle control, cell cycle inhibitors alone, anti–PD-1 antibody alone, or combined treatment of cell cycle inhibitors with anti–PD-1 antibody were administrated to *Trp53^KO^ c-Myc^OE^* HCC tumor–bearing C57BL/6N mice after HDTVi. Paclitaxel and anti–PD-1 antibody were administrated 19 days and 21 days after HDTVi, respectively (*n* = 11 mice/group). AZD1152 and anti–PD-1 antibody were administrated 16 days and 30 days after HDTVi, respectively (*n* = 10 mice/group). Palbociclib and anti–PD-1 antibody were administrated 18 days and 21 days after HDTVi, respectively, continuously (*n* = 12 mice/group). (**D**) Survival curves of *Sting* wild-type (WT) and *Sting*-knockout (KO) HCC-bearing mice with vehicle control or paclitaxel treatment administrated 22 days after HDTVi (*n* = 12 mice/group). The percentage of surviving mice was determined over treatment time. Log-rank (Mantel-Cox) test. *P < 0.05, **P < 0.01, ***P < 0.001, ****P < 0.0001.

SASP consisting of chemokines, cytokines, proteases, and growth factors are believed to be pro-inflammatory and initiate immune response (35–37). In order to verify if cell cycle inhibitors generally induced SASP expression–triggered immune response, C57BL/6N immunocompetent mice were used for in vivo experiments. HCC was induced in mice using the hydrodynamic tail vein injection (HDTVi) model with the CRISPR/Cas9 system to knock out transformation-related protein 53 (*Trp53*) and with the Sleeping Beauty transposon system to overexpress *c-Myc* (*Trp53^KO^ c-Myc^OE^*). The IF staining of glucose transporter 1 (Glut1), the hypoxia marker, in *Trp53^KO^ c-Myc^OE^* tumor tissue verified the hypoxic environment in tumor (Supplemental Figure 9A). AZD1152 and GSK1070916 are both ATP-competitive aurora kinase B inhibitors. Additionally, AZD1152 has been studied clinically, with 15 trials in phases I, II, and III to date, in various cancer models (38). Therefore, we performed the drug treatment with AZD1152 for in vivo experiments. The mice were administrated with paclitaxel, AZD1152, or palbociclib 3 weeks after HDTVi. All 3 cell cycle inhibitors significantly reduced the HCC tumor size without causing weight loss or adverse effect in mice (Figure 5A and Supplemental Figure 9B). H&E staining of HCC tissues in AZD1152 and palbociclib experiments showed that the tumor growth front of vehicle control–treated groups (Ctrl) was generally more irregular when compared with the cell cycle inhibitor–treated groups, while there was no difference between Ctrl- and inhibitor-treated groups in the paclitaxel experiment (Supplemental Figure 10A and Supplemental Tables 1–3). Further, venous invasion was only present in Ctrl groups in all 3 (paclitaxel, AZD1152, palbociclib) experiments (Supplemental Figure 10B and Supplemental Tables 1–3). The IHC staining of the mouse HCC tumors clearly uncovered the profound increase of CD4^+^ T cell, CD8^+^ T cell, and NK cell staining within the tumor boundary (Figure 5B and Supplemental Figure 11). In addition, these cell cycle inhibitors upregulated a panel of senescence markers and SASPs in the tumor core after treatment, as shown by the RNA-Seq data of mouse tumor tissues (Supplemental Figure 12). In general, we observed a differential expression of the senescence signatures in different treatment groups. However, the trend of induction of SASP was consistently observed in mice after paclitaxel/palbociclib/AZD1152 treatments. These indicated the cell cycle inhibitors promoted the infiltration of both innate immune cells, i.e., NK cells, and adaptive immune cells, i.e., CD4^+^ and CD8^+^ T cells, into the tumor core. Their cytotoxic activities also contributed to cancer cell clearance and the consequential reduction of tumor sizes. The activation of immune surveillance in tumor provided it with a favorable condition to combine cell cycle inhibitor treatment with immunotherapy to fully unleash the potential of immune cells for cancer clearance. Therefore, an ICI, anti–PD-1, was administrated in combination with the cell cycle inhibitors in C57BL/6N mice with HDTVi-induced *Trp53^KO^ c-Myc^OE^* HCC. The survival results illustrated minimal differences in term of OS between the untreated and single treatments. However, the combo treatment of paclitaxel plus ICI arm notably extended OS of mice bearing HCC. AZD1152 and palbociclib plus ICI arm tended to improve OS of the mice (Figure 5C and Supplemental Figure 9C). This further verified the potential of combination treatment of a cell cycle inhibitor with ICI in combating HCC. We observed that paclitaxel extended the survival of mice with *Sting*^WT^ HCC, and the trend was less clear in *Sting*^KO^ HCC (Figure 5D and Supplemental Figure 9D). It is worth noting that this set of data on the effects of paclitaxel on *Sting*^WT^ HCC and *Sting*^KO^ HCC has not reached statistical significance. Therefore, it could not provide direct evidence that cell cycle inhibitors activate STING. However, the data imply that cell cycle inhibitors elicited antitumor effects partially through STING.

## Discussion

### Boosting the efficacy of current cell cycle inhibitors in HCC cancer treatment.

Cell cycle inhibitors are potent chemotherapeutic drugs toward various cancer types except HCC. There are many cell cycle inhibitors available in the pharmaceutical market targeting different phases of the cell cycle.

**Figure 6:**
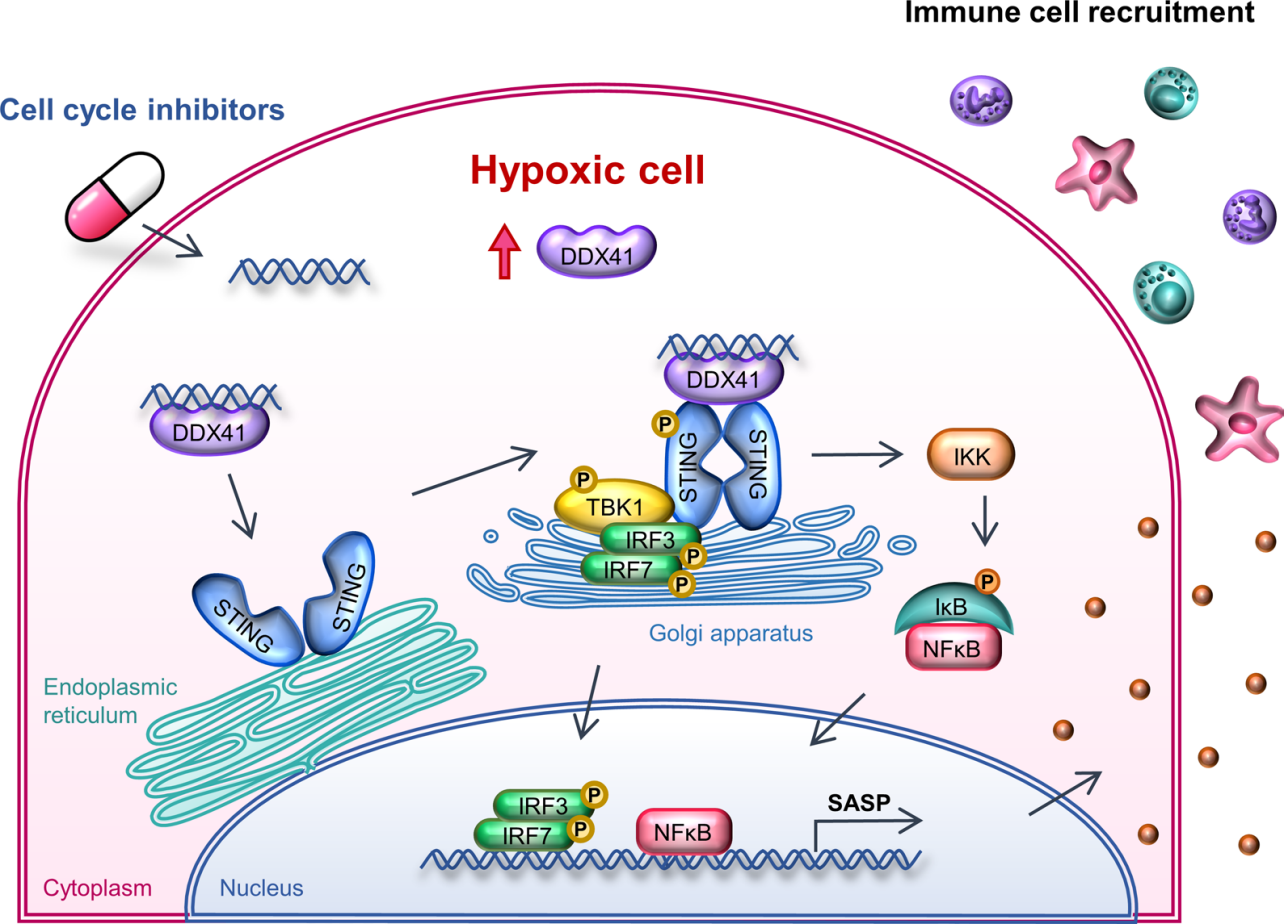
Cell cycle inhibitors trigger immune response via the DDX41/STING pathway. Schematic diagram showing cell cycle inhibitors trigger immune response via the DDX41/STING pathway. HCC tumor is highly hypoxic, which induces the upregulation of DDX41 expression in cells. Since both hypoxia and overexpression of DDX41 are detrimental to cells, cell cycle inhibitors are used to induce cytosolic DNA accumulation and trigger the activation of DDX41 as a cytosolic DNA sensor. The activated DDX41 binds to both the cytosolic DNA and STING. Upon activation, STING dimerizes and translocates from the ER to Golgi apparatus and recruits TBK1. TBK1 autophosphorylates and further phosphorylates STING and facilitates the recruitment and activation of IRF3 and IRF7. IRF3 and IRF7 form homodimer or heterodimer and translocate to nucleus for SASP expression. STING also activates IκB kinase (IKK), which then phosphorylates and inhibits the inhibitor of NF-κB (IκB). Without the binding of IκB, the NF-κB translocates to nucleus for SASP expression. The secreted SASPs recruit the infiltration of both innate and adaptive immune cells, especially NK cells, CD4^+^ T cells, and CD8^+^ T cells, to the HCC tumor core for cancer clearance. The cytotoxic potential of those infiltrated immune cells can be further enhanced by using ICIs, for example anti–PD-1 antibody, which suggests the potential of combined treatment of cell cycle inhibitors with immunotherapy.

HCC may develop resistance to the inhibitors, and some clinical trials implied that monotherapy did not work well on HCC (8–10) (NCT00497679, NCT00338182, and NCT00497731). Nonetheless, our paper demonstrated that all mechanistically distinct cell cycle inhibitors consistently have cell cycle–independent potential to be used in combination with ICIs. Apart from the canonical intrinsic effect of cell cycle arrest, senescence, and cell death, all the cell cycle inhibitors we tested simultaneously induced cytosolic DNA and triggered the DDX41/STING pathway for secretion of pro-inflammatory SASP (Figure 6). The activation of the STING pathway has been reported to promote the maturation, transition, and activation of T cells; dendritic cells; and NK cells (39). Our results demonstrated that cell cycle inhibitors recruited cytotoxic immune cells, including CD4^+^ T cells, CD8^+^ T cells, and NK cells, to infiltrate into the tumor core. Cytotoxic T lymphocytes (CTLs) and NK cells are the principal players for cancer immunosurveillance and the subsequent eradication of cancer cells during immune response (40). They both attack tumor cells in a similar manner. During direct killing, CTLs use exocytosis to deliver granzymes and pore-producing perforin and meanwhile secrete Fas ligand and TRAIL to induce cell death of target cells. For indirect killing, they express pro-inflammatory cytokines and chemokines to recruit other immune cells for tumor clearance (41–44).

The ICIs anti–programmed cell death ligand 1 (anti–PD-L1) and anti–PD-1 are aimed to overcome the tolerance of T cells toward cancer clearance. Since NK cells possess PD-1, NK cells can also be activated by anti–PD-1 therapy (45). A study showed that anti–PD-L1 and anti–PD-1 activate exhausted NK and T cells by rerouting their metabolic pathways, thereby unleashing their cytotoxicity against tumor cells (42). Here, we linked cell cycle perturbation with innate and adaptive immunity and enhanced the effect of cell cycle inhibitors by boosting the cytotoxicity of CTLs and NK cells with ICIs.

### Effect of hypoxia on cancer development.

Oxygen consumption is crucial in tumor to provide a satisfactory amount of energy by constant ATP production to support rapid proliferation, and thus tumor tends to grow near blood vessels. However, the tumor may propagate so fast that it outgrows the supply of blood and oxygen in chronic hypoxia (34). The oxygen level in liver shows a gradual decrease from the area around the hepatic artery to that around the portal vein and further to the vein (46). The metabolic functions are impaired when the median oxygen partial pressure in tumor tissue drops (47). On the one hand, hypoxia may retard growth and induce cell death by disrupting DNA repair and initiating cell cycle arrest, apoptosis, or necrosis by increasing p53; while on the other hand, it may promote growth and survival by facilitating the adaptation of cells to stress through HIF, e.g., inducing glycolysis, angiogenesis, and anaerobic metabolism, and allowing tumor cell migration and metastasis by aiding cell detachment, epithelial-mesenchymal transition, and adhesion (34, 47, 48). Although hypoxia can promote inflammation and cause immune cells’ infiltration, it rather facilitates recruitment of immune cells with pro-tumor immune subsets over antitumorigenic ones, leading to tumor evasion from immunosurveillance and tumor aggression (49–51). Besides, hypoxia was suggested to be related to the resistance toward chemotherapy and radiotherapy and was responsible for the poor therapeutic outcome (34). As a result, hypoxia may be considered a double-edged sword where it may induce cell death and suppress tumor growth but on the contrary may promote angiogenesis and support tumor growth.

We report *DDX41* as an HIF-regulated gene that is upregulated under hypoxia. Since upregulation of *DDX41* is associated with poor survival, and hypoxia is associated with chemotherapy and radiotherapy resistance and poor therapeutic outcome (34), both hypoxia and DDX41 are considered disadvantageous to patients with HCC. Cell cycle inhibitors in this situation act as an imperative juncture to twist the harmful DDX41 and detrimental hypoxia into a constructive and valuable tool to combat cancer cells. Cell cycle inhibitors, by triggering DNA accumulation in cytoplasm, activate DDX41, the cytosolic DNA sensor, which activates the STING pathway and the subsequent antitumor immune response. This makes the hypoxic tumor become more vulnerable to cell cycle inhibitor treatment and thus supports using cell cycle inhibitors in HCC therapy.

### Potential of combined treatment of cell cycle inhibitors with atezolizumab and bevacizumab.

Combination of ICI atezolizumab and anti-VEGF antibody bevacizumab is now the most effective first-line therapy for HCC (13, 15). However, antiangiogenic therapies, i.e. anti-VEGF, restrict blood vessel development and would elicit intratumor hypoxia, which favors tumor growth (52–54). Bevacizumab is expected to induce intratumor hypoxia due to its antiangiogenic function. Meanwhile, we showed that cell cycle inhibitors work best in hypoxic conditions as DDX41 was induced by HIFs, leading to increased transcription of SASP. Therefore, it is possible in the future that cell cycle inhibitors could be used in combination with atezolizumab and bevacizumab to achieve the most effective antitumor response in HCC.

We found that the effects of cell cycle inhibitors are far more than their canonical intrinsic cell cycle effect, like genome instability, ROS production, DNA damage, cytosolic accumulation, senescence, and apoptosis. They also contribute to a remarkable secondary effect by triggering SASP expression via the DDX41/STING/TBK1/IRF3/7 pathway. This leads to recruitment of both innate and adaptive cytotoxic immune cells to the tumor core for cancer surveillance and elimination. The combination treatment of cell cycle inhibitors with ICIs showed an enhanced effect against HCC, especially in the adverse hypoxic environment.

## Methods

### Sex as a biological variable.

Male mice were used in this study due to a higher incidence of liver cancers among males worldwide (1). Currently, there are no reports about differences between tumor incidence and pathologies related to the animal model regarding sex as variant involved in this study. Thus, sex was not considered as a biological variable.

### TCGA.

The RNA-Seq data of 50 pairs of human liver HCC tissue samples with their corresponding NT tissue samples were obtained from TCGA through the Broad GDAC Firehose (Broad Institute). The RNA-Seq by expectation maximization normalized count was used to show the expression levels of *TUBB*, *AURKB*, *CDK4*, *CDK6*, and *DDX41* between HCC and NT.

The overall and disease-free survival data of patients with HCC were obtained from TCGA, PanCancer Atlas via cBioPortal, and *Z* score of 0 was used as threshold to define the high and normal expression level of target genes. The survival data were determined using Kaplan-Meier method and were analyzed using log-rank (Mantel-Cox) test.

### Cell lines and culture.

Human HCC cell lines including PLC/PRF/5, Hep3B, and HepG2; human immortalized hepatocyte cell line, MIHA; and human embryonal kidney cell line 293FT were acquired from American Type Culture Collection. Human HCC cell line Huh7 was gifted by H. Nakabayshi from the School of Medicine at Hokkaido University, Sapporo, Hokkaido, Japan, and MHCC97L was gifted by Z.Y. Tang from Fudan University, Shanghai, China. CLC2, CLC4, and CLC13 were Chinese liver cancer cell lines that were gifted by Lijian Hui (Chinese Academy of Sciences, Shanghai, China) (55). All cell lines were tested to be mycoplasma free.

Huh7, PLC/PRF/5, and 293FT were grown in Dulbecco’s modified Eagle medium-high glucose (DMEM-HG) medium (Gibco, Thermo Fisher Scientific) with 1% penicillin-streptomycin (P/S; Gibco, Thermo Fisher Scientific) and 10% fetal bovine serum (FBS; Gibco, Thermo Fisher Scientific). MHCC97L, Hep3B, HepG2, and MIHA were grown in DMEM-HG medium with sodium pyruvate (Gibco, Thermo Fisher Scientific), 1% P/S, and 10% FBS. CLC2, CLC4, and CLC13 were grown in Roswell Park Memorial Institute 1640 medium (Gibco, Thermo Fisher Scientific) with 1% P/S, 10% FBS, 1× Insulin-Transferrin-Selenium-Sodium Pyruvate, and 40 μg/L recombinant human epidermal growth factor (PeproTech). All cell lines were incubated in 5% CO_2_ in a 37°C humidified incubator.

### Stable cell line establishment.

Human *DDX41*, *STING1*, *IRF7*, *RelA*, *HIF1A*, and *HIF1B* KD in cells were produced using shRNAs mediated by lentivirus (Supplemental Table 4). The oligonucleotides (Integrated DNA Technologies) containing specific shRNA targets or NTC were put into pLKO.1-puro vector (MilliporeSigma), which were then transfected into the cells and followed by puromycin selection to generate stable KD cell lines. The KD efficiency of shRNA was validated by its mRNA expression using qPCR.

Human *HIF1A*-KO cells were produced using TALEN. The specific TALE domain was put into the pTALEN v2 vector, which was then transfected into the cells and followed by clonal expansion to generate stable KO cell lines. DNA sequencing and SURVEYOR mutation detection kit (Transgenomic, Inc.) were used to validate the TALEN-induced frame-shift mutation.

### RNA extraction.

A total of 1 million cells were washed with PBS and incubated in 1 mL TRIzol reagent (Ambion, Thermo Fisher Scientific) at room temperature for 5 minutes. For RNA extraction from mouse tissues, frozen samples were homogenated in 1 mL TRIzol reagent. After adding 200 μL chloroform, the sample was shaken vigorously for 15 seconds and incubated at room temperature for 3 minutes. The sample was centrifuged for 15 minutes at 4°C at 13,572*g*. Around 500 μL of upper layer was withdrawn and mixed with 500 μL isopropanol by vortexing. After 10 minutes of incubation at room temperature, the sample was centrifuged for 10 minutes at 4°C at 13,572*g*. Supernatant was discarded and RNA pellet was washed with 1 mL 75% ethanol followed by 7,634*g* centrifugation for 5 minutes at 4°C twice. The pellet was left to air-dry and then dissolved in 40 μL ultrapure distilled water with 55°C incubation for 10 minutes. BioDrop μLITE spectrophotometer was used to measure the RNA concentration. RNA samples were stored at –80°C.

### RNA-Seq.

The RNA extracted were sent for paired-end Illumina HiSeq 2000 sequencing (Axeq Technologies). For each sample, more than 100,000 cells were sequenced, respectively. TruSeq Stranded mRNA Sample Prep Kit (Illumina) was utilized to prepare the mRNA library with poly(A)+. TopHat-Cufflinks pipeline was employed to handle the sequencing data and to express it in fragments per kilobase of transcript sequence per million mapped reads, which were then analyzed using GSEA.

### RT-qPCR.

A total of 1 μg RNA was utilized for reverse transcription using GeneAmp PCR Reagent Kit (Applied Biosystems, Thermo Fisher Scientific) according to the instructions from the manufacturer and was converted to cDNA. The cDNA samples were stored at –20°C.

Next, 1 μL of the 10-fold diluted cDNA was used for qPCR and mixed with 5 μL SYBR Green qPCR Master Mix (Applied Biosystems, Thermo Fisher Scientific), 0.2 μL 10 μM primer mix, and 3.8 μL ultrapure distilled water. Human 18S was used as a housekeeping gene. The qPCR was run under StepOnePlus Real-Time PCR system (Applied Biosystems, Thermo Fisher Scientific) at 95°C for 15 seconds and 40 cycles of 60°C for 1 minute and 6°C for 1 minute. Primers used in this study are listed (Supplemental Table 5).

### Cytosolic gDNA analysis.

Cells were collected and divided into 2 aliquots with equal cell number. For total DNA extraction, the cells were lysed in lysis buffer (25 mM EDTA, 10 mM Tris-HCl at pH 8.0, 0.5% SDS, 100 mM NaCl) and were ready for DNA extraction. For cytosolic DNA extraction, cells were lysed for 10 minutes in permeabilization buffer (50 μg/mL digitonin [Thermo Fisher Scientific], 150 mM NaCl, 2 mM EDTA, 50 mM HEPES [pH 7.4]) and then centrifuged for 3 minutes at 1,000*g*. The supernatant was transferred to a new tube and centrifuged for 10 minutes at 17,000*g*. The supernatant was collected and was ready for DNA extraction. For DNA extraction of both total and cytosolic DNA, phenol/chloroform/isoamyl alcohol (25:24:1, v/v, Invitrogen, Thermo Fisher Scientific) was used. The extracted DNA was precipitated overnight at –20°C by mixing with 7.5 M ammonium acetate, 100% ethanol, and glycogen. The DNA was washed with 70% ethanol, and the DNA pellet was left to air-dry and then dissolved in ultrapure distilled water. Both cytosolic and total gDNA were quantified using qPCR with human 18S primers.

### ChIP.

The cells were fixed in 1% (v/v) formaldehyde for 10 minutes at 37°C. A final concentration of 125 mM glycine was added to the sample and incubated for 5 minutes at 37°C. After washing with PBS, the cells were lysed in ChIP lysis buffer and subjected to sonication. The sonicated DNA fragments were first incubated overnight at 4°C with HIF-1α or HIF-1β antibodies, or IgG control, then incubated with Protein A/Salmon Sperm DNA Agarose Beads (Thermo Fisher Scientific). Buffer with different salt gradients was used to wash the beads. Then 1% SDS/NaHCO_3_ was used to elute the DNA from beads, which was then extracted using phenol-chloroform. The ChIP DNA was analyzed using RT-qPCR with primers flanking the HRE regions.

### Protein extraction and Western blotting.

For whole-cell protein extraction, the cells were washed with cold PBS and then collected in fresh cold PBS using a cell scraper. The cells were centrifuged for 2 minutes at 4°C at 1,508*g*. The cell pellet was resuspended with radioimmunoprecipitation assay buffer (50 mM Tris-HCl [pH 7.4], 1 mM EDTA, 150 mM NaCl, 0.05% w/v SDS, 0.7% v/v NP-40). For detection of STING dimer, the cell NETN lysis buffer (20 mM Tris-HCl [pH 8], 0.5 mM EDTA, 100 mM NaCl, 0.5% v/v NP-40) was used. The cells were then incubated on ice together with the cocktails of cOmplete protease inhibitor (Roche) and PhosSTOP phosphatase inhibitor (Roche) for 15 minutes. The sample was centrifuged for 15 minutes at 4°C at 13,572*g*, and the whole-cell protein lysate in the supernatant was collected. Bradford protein assay (Bio-Rad Laboratories) was used to measure the protein concentration, and BSA was used to generate the standard curve. The absorbance was measured at 595 nm using microplate reader. A total of 40 μg protein lysate was used to prepare sample lysate by mixing with 6× SDS sample loading buffer and then boiled at 95°C for 10 minutes. For detection of STING dimer, the lysate was mixed with sample loading buffer without β-mercaptoethanol.

For histone extraction, the cells were washed with cold PBS and then collected in triton extraction buffer (TEB) (0.02% w/v NaN_3_, 2 mM PMSF, PBS containing 0.5% Triton X-100 v/v) using a cell scraper. The cells were mixed gently and incubated on ice for 10 minutes. The sample was centrifuged for 10 minutes at 4°C at 500*g*, and the supernatant was discarded. The sample was washed with TEB twice and centrifuged for 10 minutes at 4°C at 500*g*. The pellet was resuspended in 0.2N HCl and incubated overnight at 4°C with rotation for histone extraction. After centrifugation for 10 minutes at 4°C at 2,500*g*, the histone protein lysate in the supernatant was collected to measure the protein concentration using Bradford protein assay. A total of 40 μg histone lysate was used to prepare sample lysate by mixing with 6× SDS sample loading buffer. The sample lysate was neutralized with NaOH and then boiled at 95°C for 10 minutes.

SDS-PAGE (Bio-Rad Laboratories) was used to separate different proteins in the sample lysate. A 10% acrylamide gel was used for whole protein lysate, and a 12% acrylamide gel was used for histone lysate. The gel was prepared using TGX FastCast premixed acrylamide solutions (Bio-Rad), N’, N’, N’, N’-tetramethylethane-1,2-diamine (TEMED), and 10% ammonium persulfate according to the manufacturer’s protocol. After gel electrophoresis separation, the proteins were transferred to a PVDF membrane (GE Healthcare, now Cytiva) by semidry Trans-Blot Turbo Transfer System (Bio-Rad) for 8 minutes at 25 V. The membrane was shaken in 5% nonfat milk with Tris-buffered saline (TBS) and 0.1% (v/v) Tween 20 (TBST) for 1 hour at room temperature. Specific primary antibody (Supplemental Table 6) was prepared in 5% nonfat milk and was shaken with the membrane for overnight at 4°C. The membrane was washed with shaking in 1× TBST at room temperature for 5 minutes 3 times and was then shaken with specific HRP-conjugated secondary antibody (Supplemental Table 6) for 2 hours at room temperature. The membrane was washed with shaking in 1× TBST at room temperature for 5 minutes 3 times and then soaked in Amersham ECL Prime Western Blotting Detection Reagent (GE Healthcare) for 1 minute at room temperature to generate chemiluminescent signals. The signal was then captured for analysis using Alliance Q9 Advanced chemiluminescence and spectral fluorescence imaging system (Uvitec).

### IF imaging.

Cells seeded on coverslips in a 6-well culture plate were washed with PBS and fixed in 4% (v/v) formaldehyde for 10 minutes at room temperature and then washed with shaking for 5 minutes 3 times with PBS. The cells were soaked in 0.5% Triton X-100 for 10 minutes at room temperature and then washed with shaking for 5 minutes 3 times with PBS. The cells were shaken in 3% BSA for 2 hours at room temperature and stained with specific primary antibody (Supplemental Table 6) overnight at 4°C, followed by washing with shaking for 5 minutes 3 times with PBS. The cells were stained with specific secondary antibody (Supplemental Table 6) for 2 hours at 4°C in the dark and washed with shaking for 5 minutes 3 times with PBS. The cells on coverslips were mounted with glass slides in ProLong Diamond Antifade mountant with DAPI (Invitrogen, Thermo Fisher Scientific). The slides were dried at 4°C in the dark overnight and then sealed with nail polish. The signal was then captured for analysis using Carl Zeiss LSM710 and LSM 880 confocal microscopes. For analysis in each experimental condition, more than 85 cells were analyzed for each experimental condition for micronuclei measurement. Two separate fields were quantified for each sample using ImageJ (NIH). The values of γ-H2A.X foci per nucleus were calculated by dividing the total number of γ-H2A.X foci by the number of cell nuclei. The average values of the number foci per nucleus for each treatment group were normalized to control group.

### IHC.

The mouse liver cancer tissue was fixed in 4% (v/v) formaldehyde overnight and then washed and kept in 70% ethanol before embedding in paraffin for slicing. The paraffin-embedded slices were dewaxed in xylene and then rinsed with 100%, 95%, and 80% ethanol. The slices were boiled for 15 minutes in 1 mM EDTA (pH 7.8) to retrieve the antigen. We used 1× TBS to rinse the slices, and the reaction was quenched by incubating the slices with 3% (v/v) H_2_O_2_ in 1× TBS for 30 minutes at room temperature. The slices were rinsed with 1× TBS and then incubated with 2× casein (Vector Laboratories) for 10 minutes at room temperature and after that stained with specific primary antibody (Supplemental Table 6) overnight at 4°C. The slices were washed with shaking in 1× TBST at room temperature for 5 minutes 4 times and was then stained with specific HRP-conjugated secondary antibody (Agilent Technologies) (Supplemental Table 6) for 30 minutes at room temperature. The slices were washed with shaking in 1× TBST at room temperature for 5 minutes 4 times and then immersed in DAB (MilliporeSigma) and H_2_O_2_ containing TBS. Once the signal developed, the slices were quenched with water. The slices were counterstained with hematoxylin, soaked in Scott’s tap water, and dehydrated in 80%, 95%, and 100% ethanol and xylene sequentially. For histological study, hematoxylin and eosin staining was used. Sections were finally mounted with coverslips for analysis.

### FC.

Cells were collected in cell staining buffer (1× PBS, 2 mM EDTA, 0.5% BSA), then fixed and permeabilized in fixation buffer (BioLegend) and permeabilization wash buffer (BioLegend), respectively. The cells were incubated with phospho-STING (41622, Cell Signaling Technology) and phospho-IRF3 (96421, Cell Signaling Technology) antibodies, and the fluorophore signals were detected by BD LSR Fortessa Analyzer (BD Biosciences) and analyzed using computer software FlowJo.

### Cell cycle profile.

The cells were synchronized using double thymidine by treating with 2 mM thymidine (Calbiochem) in FBS-free culture medium for 18 hours followed by releasing in full culture medium for 8 hours and then treating with 2 mM thymidine in FBS-free culture medium for 16 hours. MHCC97L cells were released in full culture medium with or without drug treatment for 12, 24, 36, 48, and 60 hours. For CLC4, cells were treated with cell cycle inhibitors for 24, 48, and 72 hours. Medium and drugs were replenished every other day. The cells were collected and fixed with 70% (v/v) ethanol at 4°C overnight. The cells were then stained with 50 μg/mL PI (MilliporeSigma) and 20 μg/mL RNase A (Invitrogen, Thermo Fisher Scientific) at 37°C for 10 minutes in the dark. The fluorophore signals were detected by LSR Fortessa Analyzer (BD Biosciences) and analyzed using computer software FlowJo.

### Apoptosis assay.

The cells together with the cell culture supernatant were collected and washed with PBS. Annexin V-FITC Kit (MBL International) was used to stain the cells according to manufacturer’s instructions. In brief, the cells were stained with annexin V binding buffer, annexin V-FITC, and PI for 15 minutes at room temperature in the dark. The fluorophore signals were detected by LSR Fortessa Analyzer and analyzed using computer software FlowJo.

### ROS quantification.

The cells were collected and washed with cold PBS and centrifuged for 1 minute at 4°C at 848*g*. The cells were stained on ice with 2 μM chloromethyl-20,70-dichlorodihydrofluorescein diacetate (CM-H_2_DCFDA; Life Technologies, Thermo Fisher Scientific) in the dark. The fluorophore signals were detected by LSR Fortessa Analyzer and analyzed using computer software FlowJo.

### ELISA.

Human CCL2/MCP-1 Quantikine ELISA Kit (R&D Systems, Bio-Techne) was used to quantify the amount of CCL2 secreted by cells in medium according to the manufacturer’s protocol. In brief, the cell culture supernatant was collected and filtered with a 0.45 μm filter to remove cell debris. The filtered supernatant was added to a 96-well ELISA plate and incubated for 2 hours at room temperature. Each well was washed with wash buffer 3 times and incubated with human MCP-1 conjugate for 1 hour at room temperature and then washed 3 times again. Substrate solution was added and incubated for 30 minutes in the dark at room temperature, followed by the addition of stop solution. The optical density was measured at 450 nm using a microplate reader with wavelength correction at 570 nm. Four-parameter logistic curve fit was used to generate the standard curve for data analysis.

### SA-β-Gal activity assay.

A senescence β-galactosidase staining kit (Cell Signaling Technology) was used to detect the activity of β-galactosidase in senescent cells according to the manufacturer’s protocol. In brief, the cells were washed with PBS and fixed with fixative solution for 15 minutes at room temperature. The cells were then washed with PBS and stained with β-galactosidase staining solution at pH 6 overnight at 37°C in a dry incubator in the dark. The cell images were captured and analyzed using an inverted microscope (ECLIPSE Ts2, Nikon).

### HDTVi.

Male C57BL/6N mice with age of 8 to 10 weeks provided by the Centre for Comparative Medicine Research (CCMR) in the University of Hong Kong were used for HDTVi. A mixture of plasmid DNA containing *Trp53*-KO plasmid, *c-Myc*–OE plasmid, and Sleeping Beauty transposon plasmid were prepared in saline (*Trp53^KO^ c-Myc^OE^*). For the *Sting*-KO HCC model, *Trp53-Sting*-KO plasmid was used instead of *Trp53*-KO plasmid. The plasmid mixture of volume equaling 10% mice body weight was injected within 7 seconds into the lateral tail vein of mice. The drug treatment was started 2 to 3 weeks after HDTVi, and the mice were weighed 2 to 3 times a week. At the experiment endpoint, the liver tumors were harvested and weighed. Male mice were used in this study due to a higher incidence of liver cancers among males worldwide (1). Currently, there are no reports regarding differences between tumor incidence and pathologies related to the animal model involved in this study.

### Drug treatment.

For the in vitro assay, 10 nM paclitaxel (P-9600, LC Laboratories), 50 nM GSK1070916 (HY-70044, MedChemExpress), 500 nM AZD1152-HQPA (SML0268, MilliporeSigma), 10 μM palbociclib (P-7788, LC Laboratories), and 50 μM ZVF (S7023, Selleckchem) were used unless otherwise specified. All the drugs were reconstituted in DMSO.

For in vivo drug administration, paclitaxel in DMSO was diluted in 60% PEG (MilliporeSigma) and was administered via intraperitoneal (IP) injection twice a week at 10 mg/kg. AZD1152-HQPA in DMSO was diluted in 40% PEG and was administered via IP injection every 3 days at 10 mg/kg. Palbociclib was dissolved in ultrapure water and was administered orally daily at 75 mg/kg. Anti–PD-1 antibody (BE0146, BP0146, BioXCell) was diluted in saline and was administered via IP injection twice a week for 3 weeks at 10 mg/kg unless otherwise specified.

### Statistics.

All statistical analysis was determined using GraphPad Prism 8.0 software. The data were demonstrated as mean ± SD, and 2-tailed Student’s *t* test, Wilcoxon’s signed-rank test, Kaplan-Meier test, log-rank (Mantel-Cox) test, and 1-way and 2-way ANOVA with Bonferroni’s correction were used for analysis when appropriate. Statistically significant *P* value was defined at less than 0.05.

### Study approval.

All animal experimental procedures were reviewed and approved by the CCMR, which is an Association for Assessment and Accreditation of Laboratory Animals International–accredited service center of the Faculty of Medicine in the University of Hong Kong (CULATR protocol 4889-18).

### Data availability.

Patient data from online TCGA database were retrieved from http://www.cbioportal.org/ Next-generation sequencing data are available in National Center for Biotechnology public repositories, the Sequence Read Archive system (PRJNA1153299, https://dataview.ncbi.nlm.nih.gov/object/PRJNA1153299?reviewer=fhc65p0if6up4cihq1thsteb6c; PRJNA1153321, https://dataview.ncbi.nlm.nih.gov/object/PRJNA1153321?reviewer=n3a4g4bddaejamlb3s092t73fb). The code utilized in this study is proprietary in-house code and is available upon request.

## Author contributions

The conception and design of the study were contributed by PYW, CYKC, HDGX, and CCLW. Acquisition, analysis, and interpretation of data were performed by PYW, CYKC, HDGX, CCG, JWSC, APWT, MSZ, and YZ. Drafting of the manuscript was done by PYW, CYKC, HDGX, and CCLW. Final approval of the version submitted for publication was given by CCLW.

## Supplementary Material

Supplemental data

Unedited blot and gel images

Supporting data values

## Version 1 10/10/2024

In-Press Preview

## Version 2 11/22/2024

Electronic publication
