# Supplementary material for: Cell cycle inhibitors activate the hypoxia-induced DDX41/STING pathway to mediate antitumor immune response in liver cancer
Source: JCI Insight. 2024 Nov 22;9(22):e170532. doi: 10.1172/jci.insight.170532 (PMC11601891; doi:10.1172/jci.insight.170532)
Supplement: Supplemental data [file jciinsight-9-170532-s306.pdf]

**Supplemental material**

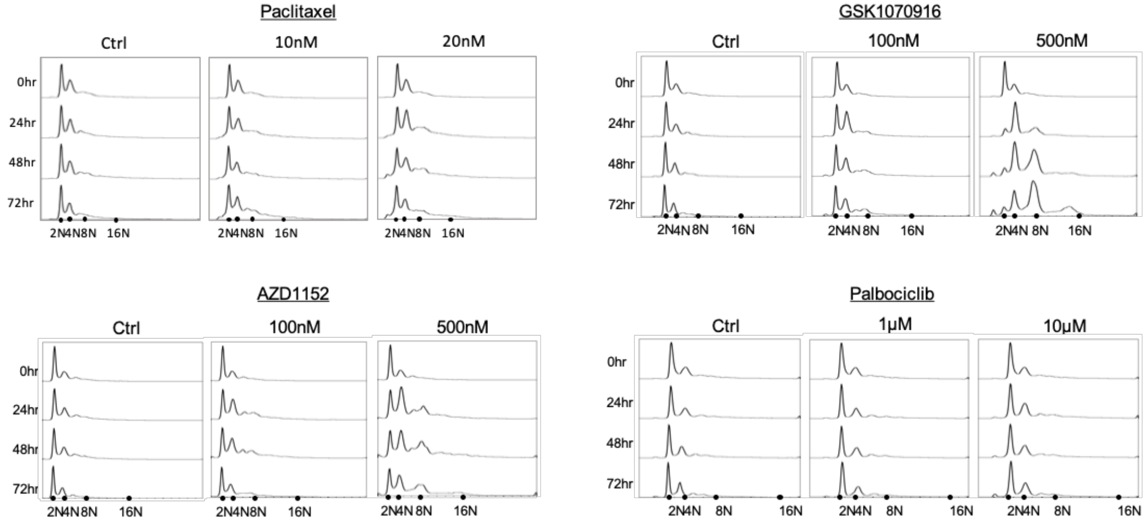


**Supplementary figure 1. Cell cycle inhibitors led to genome instability in HCC cells**

HCC cell line CLC4 were first synchronized at G1/S boundary with double thymidine block. Upon release from the second thymidine block at 0 hour time point, synchronized HCC cells were then treated with indicated concentration of the cell cycle inhibitors. Cell population were harvested at a 24-hour interval, up to 72 hours. Number of cells analyzed in each treatment (*N*) ≥ 10,000.

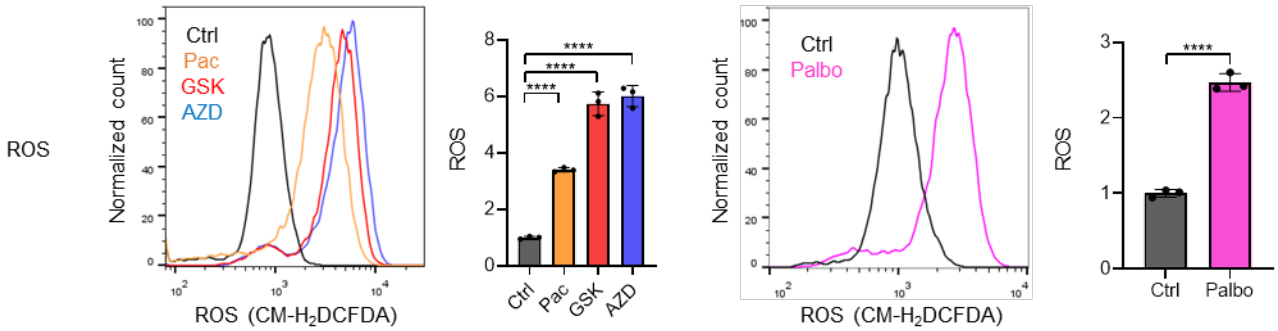


**Supplementary figure 2. Cell cycle inhibitors induced cellular oxidative stress in HCC cells**

MHCC97L cells were treated with cell cycle inhibitors for 48 hours. The intracellular ROS level was detected using CM-$H_2$DCFDA. The ROS level in inhibitor-treated cells was normalized to that in control cells (n=3/group). Scatter dot plot: mean with SD. Student's t test. * $P < 0.05$, ** $P < 0.01$, *** $P < 0.001$, **** $P < 0.0001$.

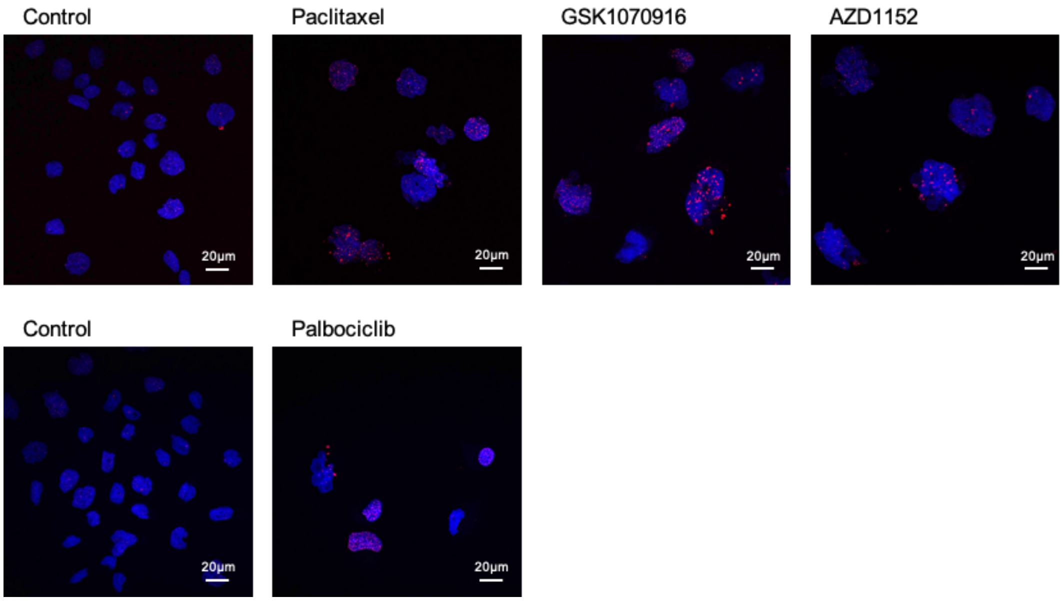


**Supplementary figure 3. Representative images showing DNA damage in cell cycle inhibitors-treated HCC cells**

MHCC97L cells were treated with cell cycle inhibitors for 48 hours and subjected to IF staining at 40x magnification. DNA damage was detected using γ-H2A.X. Blue: DAPI; Red: γ-H2A.X. Number of cells analyzed in each treatment (*N*) ≥ 85. Scale: 20 μm.

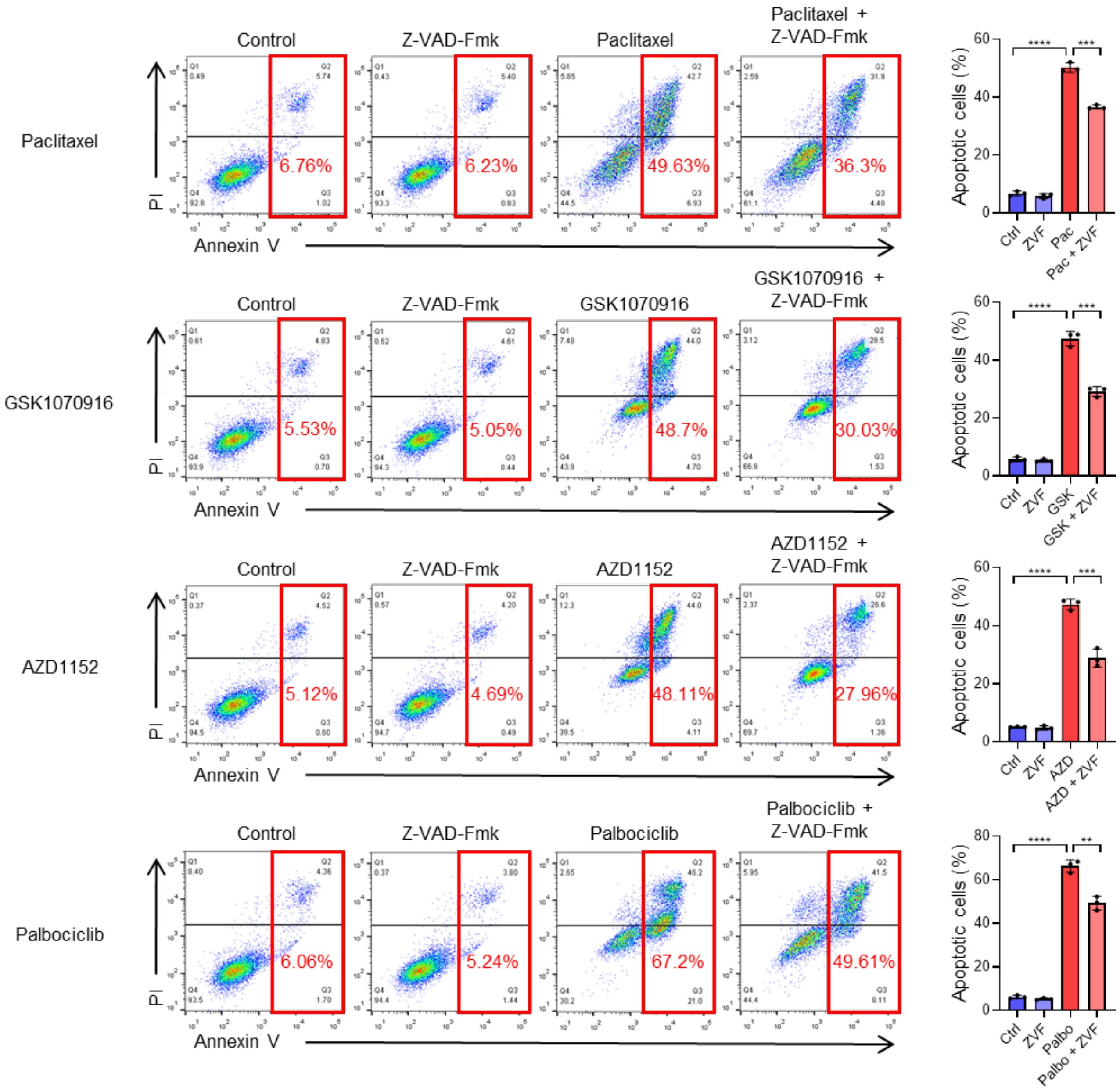


**Supplementary figure 4. Cell cycle inhibitors induced apoptosis**

MHCC97L cells were treated with cell cycle inhibitors alone, ZVF alone or co-treated with both cell cycle inhibitors and ZVF for 72 hours. Apoptosis was detected using Annexin V and PI staining and analyzed using flow cytometry. The percentage of apoptotic cells was determined by both Annexin V single positive staining cells and PI-Annexin V double

positive staining cells (n=3/group). Scatter dot plot: mean with SD. Student's t test. * $P < 0.05$, ** $P < 0.01$, *** $P < 0.001$, **** $P < 0.0001$.

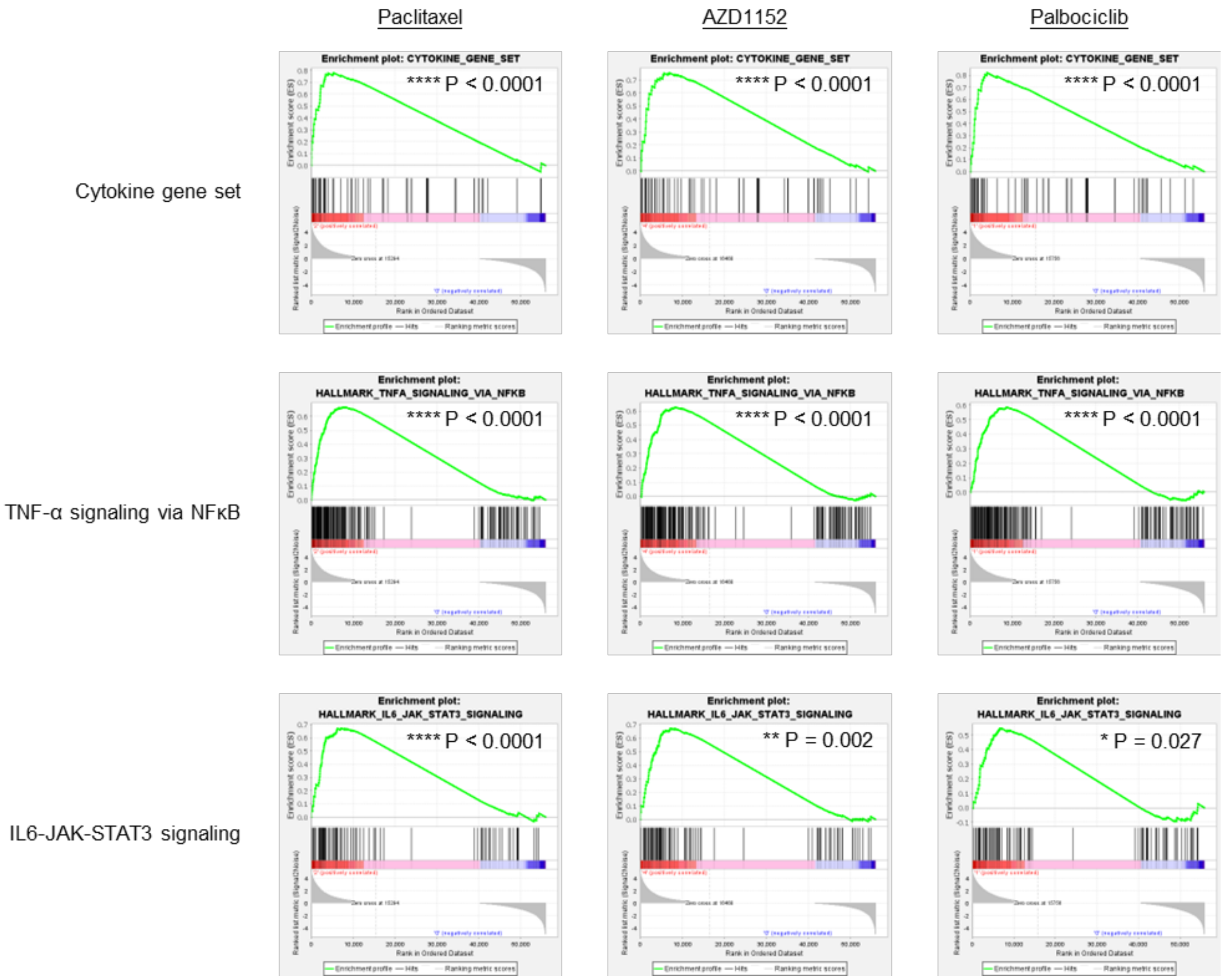


**Supplementary figure 5. Pathways enriched in cell cycle inhibitors-treated HCC cells**

MHCC97L cells were treated with cell cycle inhibitors for 96 hours. RNA was extracted and prepared for RNA sequencing. RNA sequencing data was used to analyze the enrichment of cytokine gene set, TNF-α signaling via NFκB and IL6-JAK-STAT3 signaling using GSEA. Number of cells analyzed in each treatment (*N*) ≥ 1e5. Student's t test. * P < 0.05, ** P < 0.01, *** P < 0.001, **** P < 0.0001.

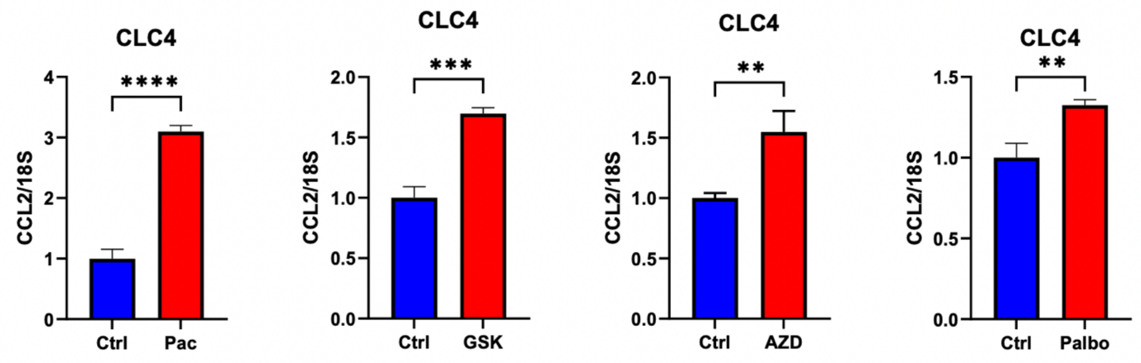


**Supplementary figure 6. Cell cycle inhibitors induced SASP expression in HCC cell line**

After treatment with cell cycle inhibitors for 192 hours, RNA was extracted HCC cell line CLC4 and mRNA expression of the target gene *CCL2* was determined using RT-qPCR and normalized to housekeeping gene *18S* (n=3/group). Column bar graph: mean with SD. Student's t test. * $P < 0.05$, ** $P < 0.01$, *** $P < 0.001$, **** $P < 0.0001$.

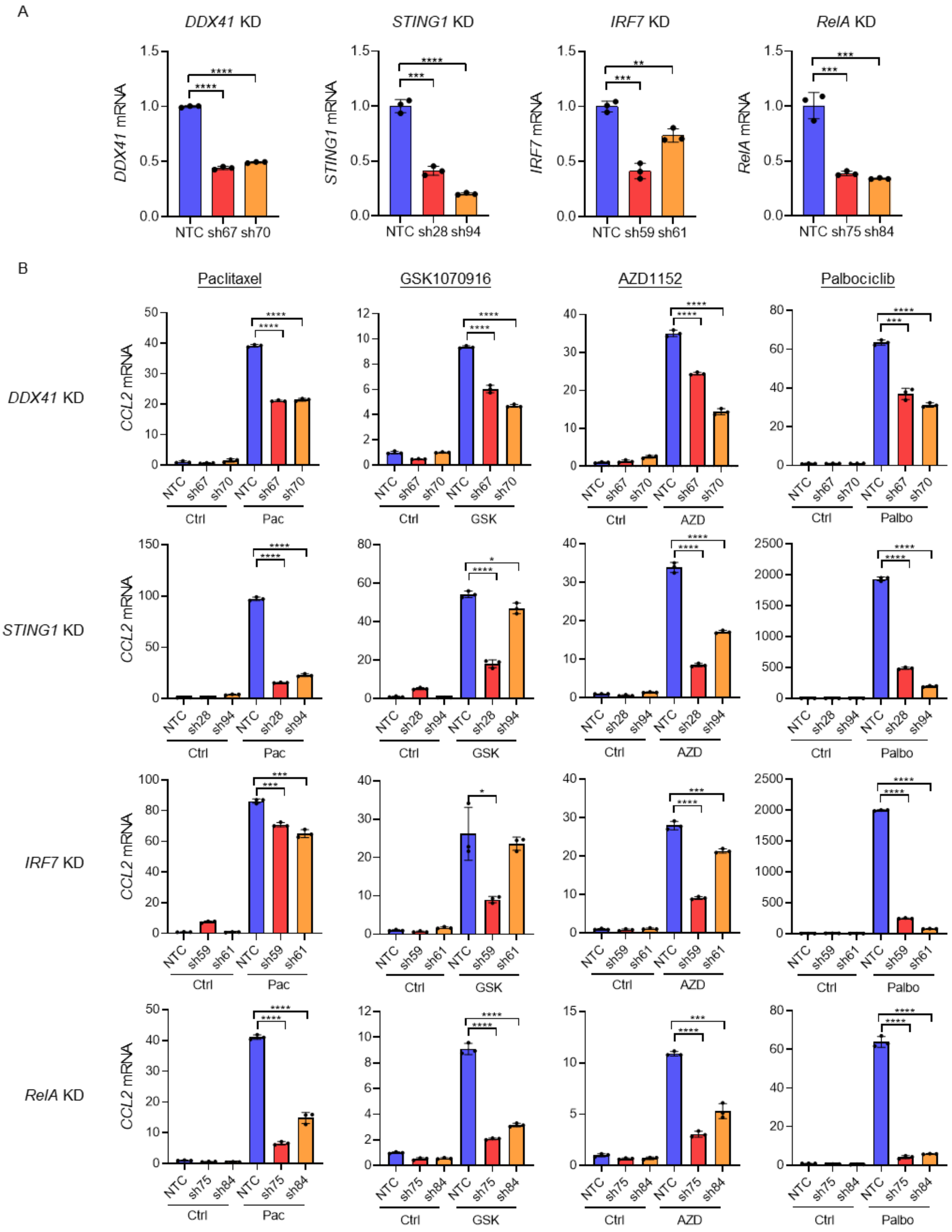
A
DDX41 KD
STING1 KD
IRF7 KD
RelA KD
DDX41 mRNA
STING1 mRNA
IRF7 mRNA
RelA mRNA
NTC sh67 sh70
NTC sh28 sh94
NTC sh59 sh61
NTC sh75 sh84
B
Paclitaxel
GSK1070916
AZD1152
Palbociclib
DDX41 KD
STING1 KD
IRF7 KD
RelA KD
CCL2 mRNA
Ctrl
Pac
GSK
AZD
Palbo

**Supplementary figure 7. Cell cycle inhibitors induced SASP expression via DDX41-STING pathway**

**A.** Efficiency of KD of genes. *DDX41*, *STING 1*, *IRF7* and *RelA* KD clones were generated in MHCC97L cells using shRNA. The cells from each KD clones were collected and RNA was extracted. The relative mRNA expression of the target gene was determined using RT-qPCR and normalized to housekeeping gene *18S*. The KD efficiency was determined by normalizing the target gene expression in KD clone to that in non-targeting control (NTC) clone (n=3/group). **B.** MHCC97L stable KD clones were treated with cell cycle inhibitors for 96 hours. The RNA was extracted and mRNA expression of *CCL2* was determined using RT-qPCR (n=3/group). Scatter dot plot: mean with SD. Student's t test. * $P < 0.05$, ** $P < 0.01$, *** $P < 0.001$, **** $P < 0.0001$.

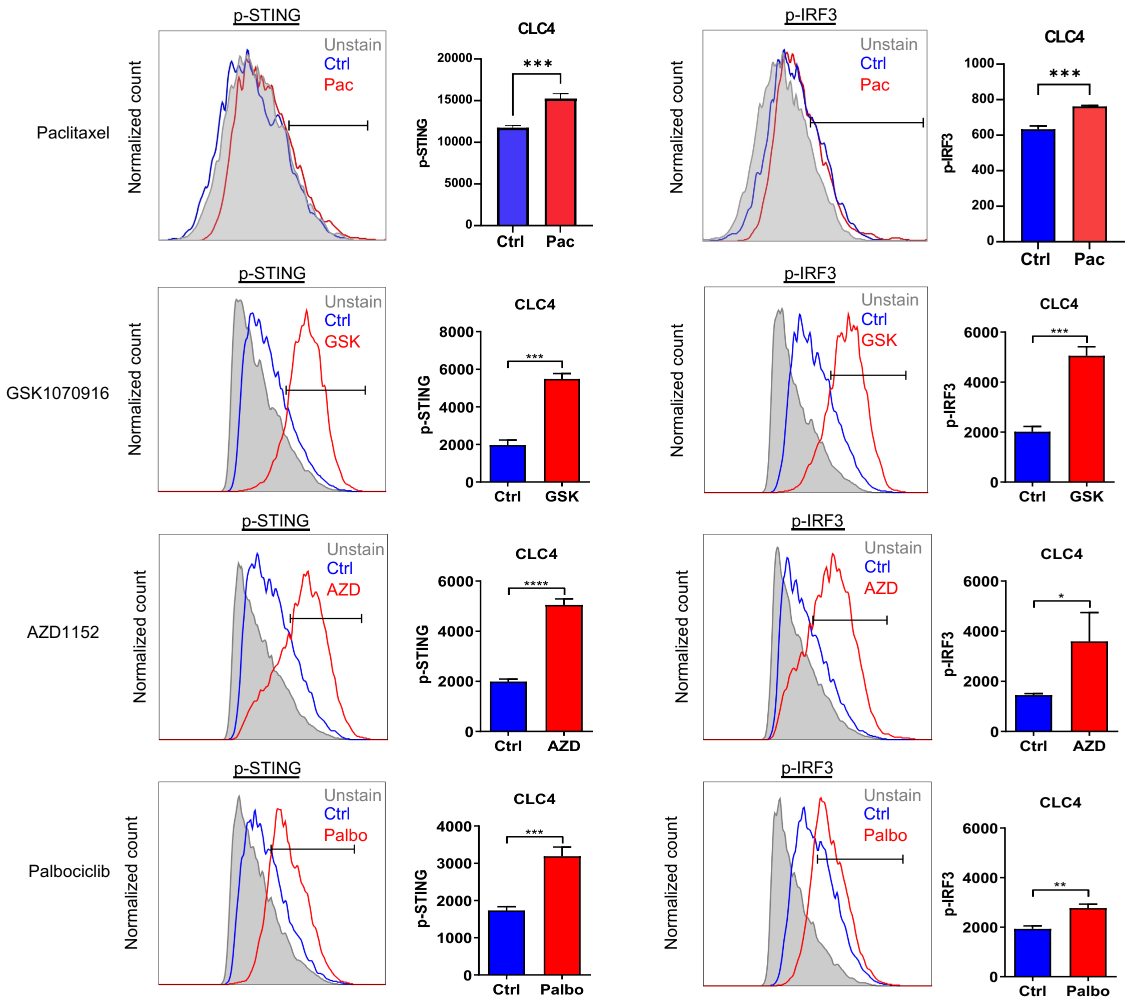


**Supplementary figure 8. Cell cycle inhibitors led to genome instability in HCC cells**

HCC cell line CLC4 were treated with 10 nM Paclitaxel for 192 hours, 100 nM GSK1070916, 100 nM AZD1152 or 10 μM Palbociclib for 120 hours. Upon collection, cells were stained for the phospho-STING (p-STING) and phospho-IRF3 (p-IRF3) with specific antibodies, hence, detected and analyzed using flow cytometry (n=3/group). Column bar graph: mean with SD. Student's t test. * $P < 0.05$, ** $P < 0.01$, *** $P < 0.001$, **** $P < 0.0001$.

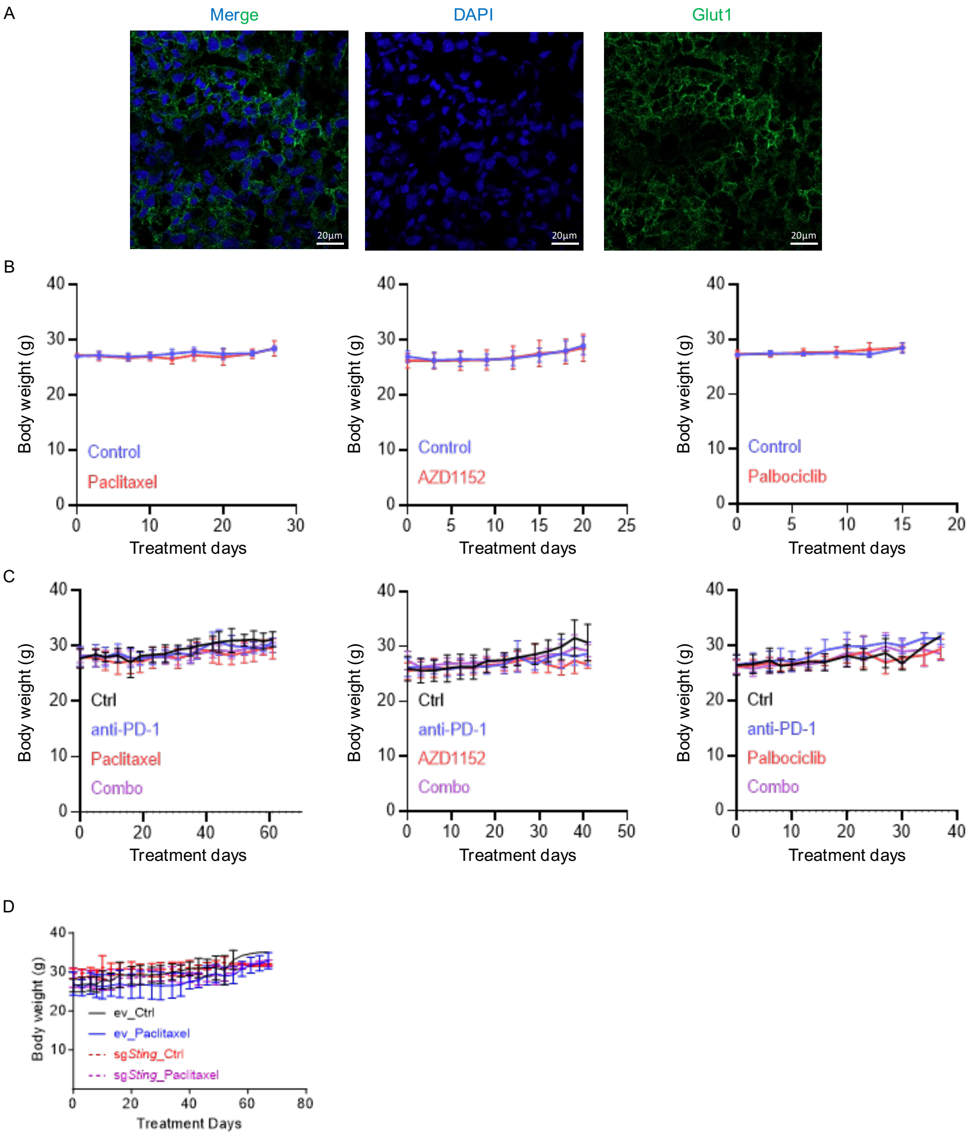


**Supplementary figure 9. Cell cycle inhibitors treatment did not cause weight loss in mice with hypoxic HCC tumors**

**A.** Hypoxia in *Trp53*$^{KO}$/*c-Myc*$^{OE}$ HCC tumor-bearing C57BL/6N mice was detected using glut1 in IF staining. Blue: DAPI; Green: Glut1. Scale: 20 μm. **B - C.** The body weight of mice treated with **B.** single cell cycle inhibitor treatment, or **C.** combination treatment of cell cycle inhibitor with anti-PD-1 were measured regularly during the treatment period. **D.**

The body weight of mice treated with vehicle control (Ctrl) or Paclitaxel treatment. Mean and error: SD**.**

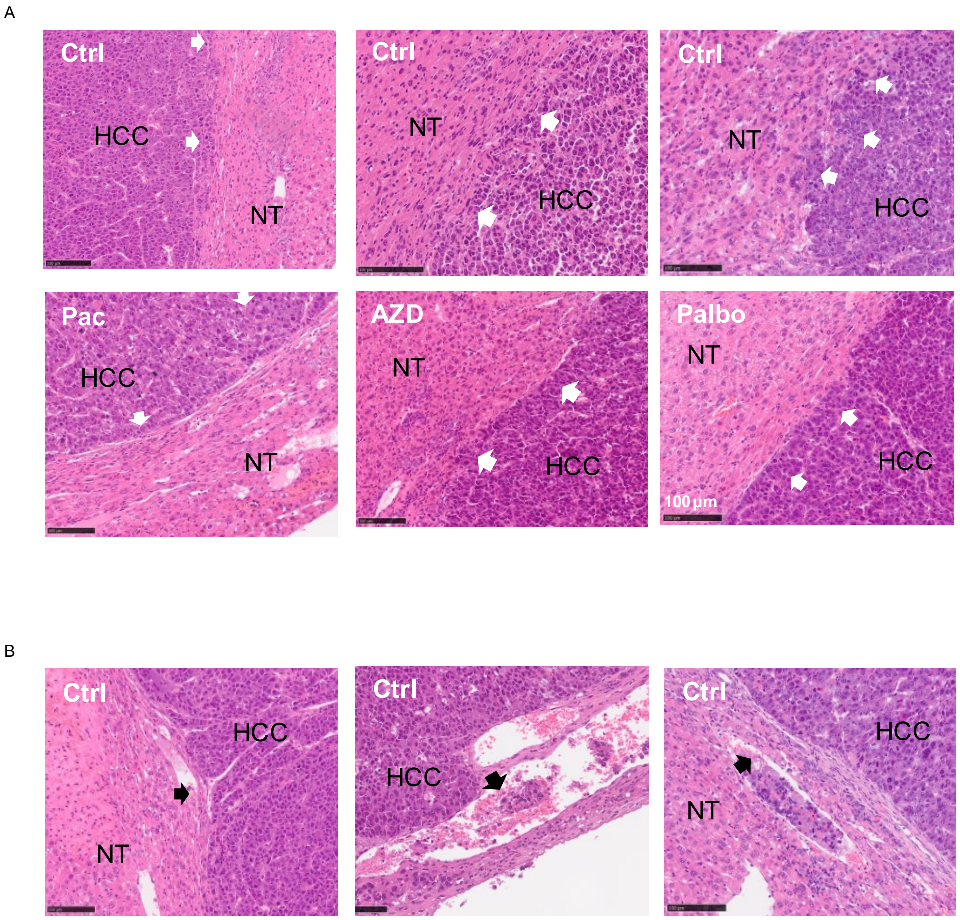


**Supplementary figure 10. H&E staining of livers in HCC bearing mice treated with vehicle control (Ctrl) and cell cycle inhibitors (Pac, AZD, Palbo)**

Representative images illustrating (A) HCC and non-tumorous (NT) tissues boundary (B) presence of venous invasion (left: Paclitaxel, middle: AZD1152, right: Palbociclib experiments). **A.** White arrows indicate HCC and NT tissues boundary. **B.** Black arrows indicate venous invasion.

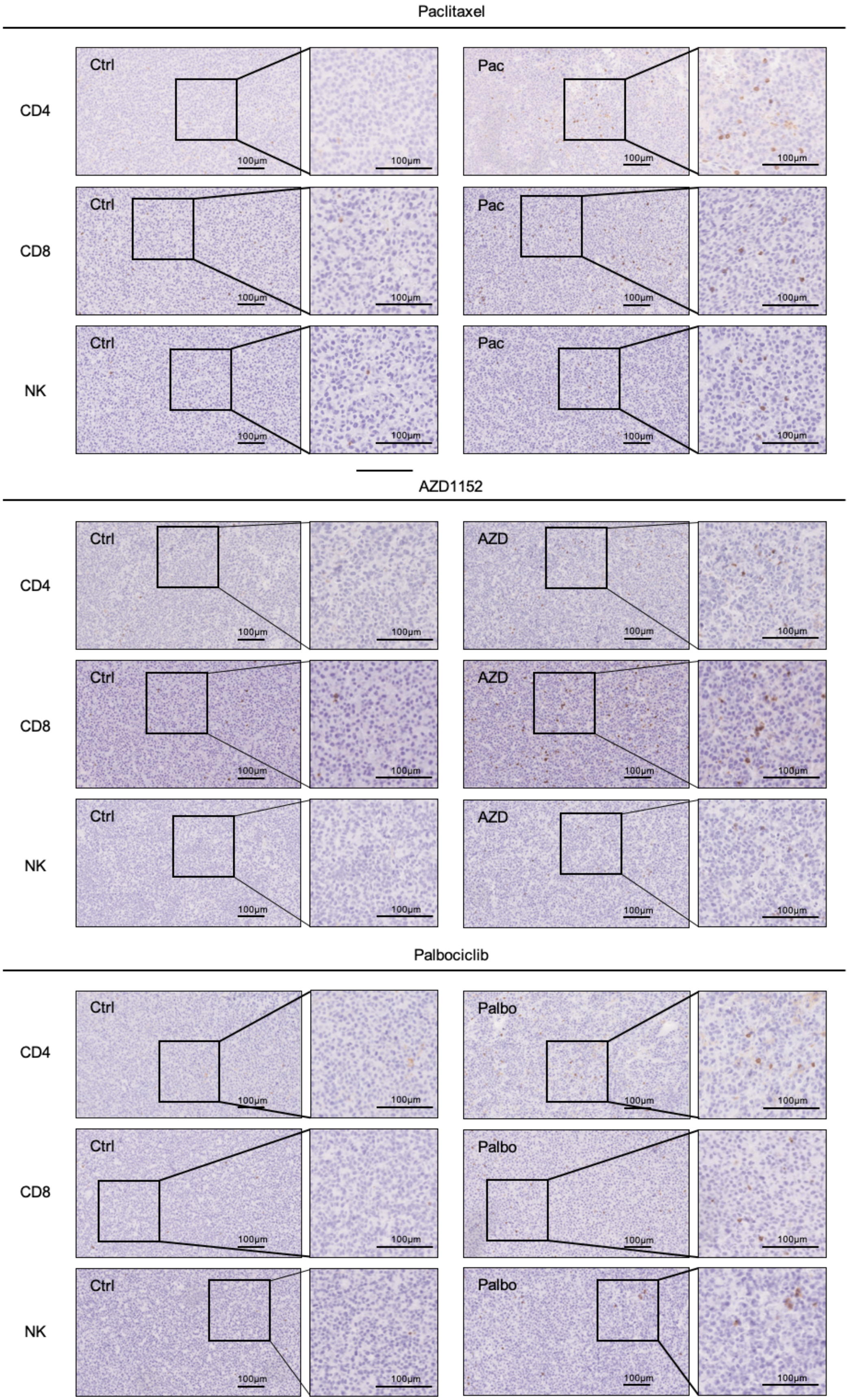
Paclitaxel
CD4
CD8
NK
Ctrl
Pac
100μm
AZD1152
AZD
Palbociclib
Palbo

**Supplementary figure 11. Representative images showing the IHC staining of CD4 T cells, CD8 T cells and NK cells**

Cell cycle inhibitors were administrated to *Trp53*$^{KO}$/*c-Myc*$^{OE}$ HCC tumor-bearing C57BL/6N mice. The tumors were harvested and prepared for paraffin-embedded slices (The plots were analyzed with 3 sections from each mouse, in total 15 sections from 5 mice). CD4 T cells, CD8 T cells and NK cells were detected using mouse CD4, CD8α and KLrb1c/CD161c antibodies in IHC staining. Scale: 100 μm.

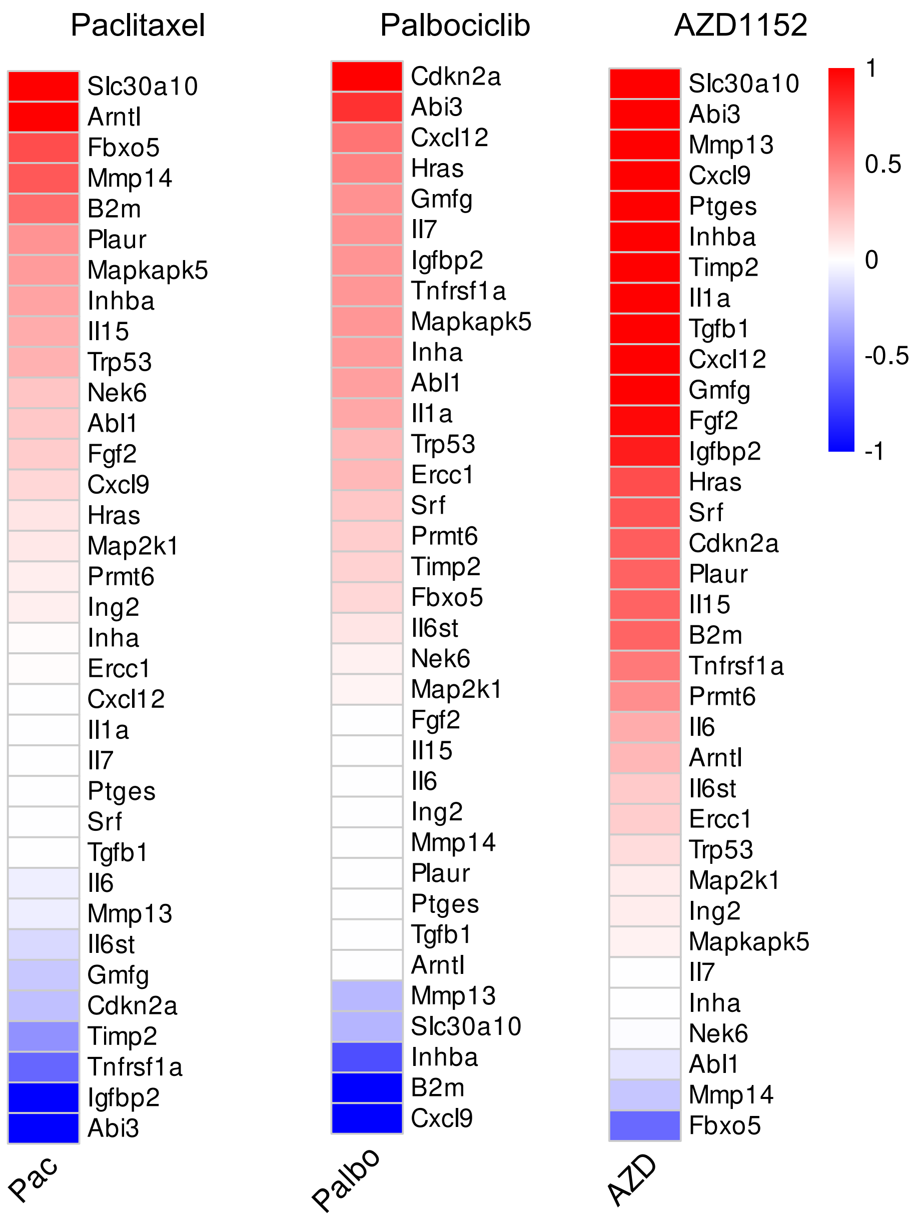


**Supplementary figure 12. Senescence markers and SASPs in cell cycle inhibitors-treated mouse HCC.**

Heatmap showing results of RNA sequencing performed on cell cycle inhibitors-treated mouse HCC tissues. Drug treated samples were compared with vehicle control treated counterparts. Log2 fold change of gene expressions is shown.

## Tables

| Treatment group | Mouse_id | Growth Front | Venous Invasion |
|---|---|---|---|
| Ctrl | C1 | mostly irregular | venous invasion |
| | C2 | Partial irregular, partial regular | NA |
| | C3 | No tumor boundary could be seen | |
| | C4 | mostly irregular | NA |
| | C5 | Irregular | Multiple Venous Invasion |
| | C6 | mostly irregular | NA |
| | C7 | Partial irregular, partial regular | NA |
| | C8 | Irregular | NA |
| Paclitaxel | P1 | Irregular | NA |
| | P2 | mostly regular | NA |
| | P3 | mostly irregular | NA |
| | P4 | Partial irregular, partial regular | NA |
| | P5 | Partial irregular, partial regular | NA |
| | P6 | Mostly regular | NA |
| | P7 | Mostly irregular | NA |
| | P8 | mostly irregular | NA |

**Supplementary Table 1. Analysis of H&E staining in vehicle control (Ctrl) treated and Paclitaxel treated mice.**

| Treatment group | Mouse_id | Growth Front | Venous Invasion |
|---|---|---|---|
| Ctrl | C1 | Partially regular, partially irregular | Multiple Venous Invasion |
| | C2 | Irregular | NA |
| | C3 | Irregular | NA |
| | C4 | Irregular | NA |
| | C5 | Irregular | NA |
| | C6 | Irregular | Venous Invasion |
| | C7 | regular | NA |
| | C8 | regular | NA |
| AZD | A1 | Irregular | NA |
| | A2 | regular | NA |
| | A3 | regular | NA |
| | A4 | regular | NA |
| | A5 | regular | NA |
| | A6 | regular | NA |
| | A7 | regular | NA |
| | A9 | Partially regular, partially irregular | NA |

**Supplementary Table 2. Analysis of H&E staining in vehicle control (Ctrl) treated and AZD1152 treated mice.**

| Treatment group | Mouse_id | Growth Front | Venous Invasion |
|---|---|---|---|
| Ctrl | C1 | Partially regular, partially irregular | NA |
| | C2 | Partially regular, partially irregular | Venous Invasion |
| | C3 | Irregular | NA |
| | C4 | Partially regular, partially irregular | NA |
| | C5 | Irregular | NA |
| | C6 | Irregular | NA |
| | C7 | Irregular | NA |
| | C8 | Partially regular, partially irregular | NA |
| Palbo | Palbo1 | regular | NA |
| | Palbo2 | Partially irregular | NA |
| | Palbo3 | Irregular | NA |
| | Palbo4 | regular | NA |
| | Palbo5 | regular | NA |
| | Palbo6 | regular | NA |
| | Palbo7 | regular | NA |

**Supplementary Table 3. Analysis of H&E staining in vehicle control (Ctrl) treated and Palbociclib treated mice.**

| shRNA | Target sequence |
|---|---|
| HIF1A | GTTACGTTCCTTCGATCAG |
| HIF1B-97 | GGCTCAAGGAGATCGTTTATT |
| HIF1B-98 | ACTAGGTCCCACAGCTAATTT |
| DDX41-67 | GCCACTACCTTCATCAACAAA |
| DDX41-70 | CGCCACTACCTTCATCAACAA |
| STING1-28 | CCAACATTCGCTTCCTGGATA |
| STING1-94 | GTTTACAGCAACAGCATCTAT |
| IRF7-59 | GCTGGACGTGACCATCATGTA |
| IRF7-61 | CTGTTCGGAGAGTGGCTCCTT |
| RelA-75 | GGAGTACCCTGAGGCTATAAC |
| RelA-84 | CGGATTGAGGAGAAACGTAAA |

**Supplementary Table 4. List of shRNA target sequences**

| Target gene | 5'-3' sequence |
|---|---|
| 18S Forward | GAGGATGAGGTGGAACGTGT |
| 18S Reverse | AGAAGTGACGCAGCCCTCTA |
| CCL2 Forward | CAAGTGTCCCAAAGAAGCTGTG |
| CCL2 Reverse | GGTTTGCTTGTCCAGGTGGT |
| DDX41 Forward | ATCCCACCACCCATCAAGAG |
| DDX41 Reverse | TGTCACGGCCAGATAGAATGG |
| STING1 Forward | CATGGGCTGGCATGGTCATA |
| STING1 Reverse | CCCCGTAGCAGGTTGTTGTA |
| IRF7 Forward | GCTCCCCACGCTATACCATCTA |
| IRF7 Reverse | AGCCAGGGTTCCAGCTTCAC |
| RelA Forward | GCGAGAGGAGCACAGATACC |
| RelA Reverse | TCCCCACGCTGCTCTTCTAT |
| DDX41_HRE_536F | TCAAAACCACTGCCATCCGT |
| DDX41_HRE_536R | GGGATAAACCGCTCGACACA |
| DDX41_HRE_422/365F | TGTGTCGAGCGGTTTATCCC |
| DDX41_HRE_422/365R | CTCCCAGAGCATGGCGTCTT |
| DDX41_HRE_36/2F | CGTCGTTCGCCTCTTCACA |
| DDX41_HRE_36/2R | GGTTCCGACTCCTCCATTCTTT |

**Supplementary Table 5. List of primer sequences**

| Antibody | Supplier | Amount / Dilution | Catalogue no. | purpose |
|---|---|---|---|---|
| HIF-1α | Abcam | 5 µg | ab1 | ChIP |
| HIF-1β | Abcam | 5 µg | ab2 | ChIP |
| Mouse IgG | Santa Cruz | 5 µg | sc2762 | ChIP |
| Rabbit IgG | Invitrogen | 5 µg | 10500C | ChIP |
| DDX41 (D3F1Z) | Cell Signaling Technology | 1:1000 | 15076 | WB |
| Histone H3 | Sigma | 1:3000 | 05-928 | WB |
| STING (D2P2F) | Cell Signaling Technology | 1:1000 | 13647 | WB |
| HIF-1α | Cell Signaling Technology | 1:1000 | 3716 | WB |
| Phospho-Histone H2A.X (Ser139) | Sigma | 1:1000 | 05-636-I | WB |
| β-actin | Sigma | 1:2500 | A5316 | WB |
| Mouse IgG HRP Linked | Sigma | 1:2500 | NA931 | WB |
| Rabbit IgG HRP Linked | Sigma | 1:2500 | NA934 | WB |
| Glut 1 | Abcam | 1:200 | ab15309 | IF |
| Phospho-Histone H2A.X (Ser139) | Sigma | 1:200 | 05-636-I | IF |
| Goat anti-Rabbit IgG (H+L) Alexa Fluor 488 | Invitrogen | 1:1000 | A27304 | IF |
| Goat anti-Mouse IgG (H+L) Alexa Fluor 594 | Invitrogen | 1:600 | A11005 | IF |
| Mouse CD4 | Cell Signaling Technology | 1:100 | 25229 | IHC |

| | | | | |
|---|---|---|---|---|
| Mouse CD8α | Cell Signaling Technology | 1:200 | 98941 | IHC |
| Mouse KLrb1c/CD161c (NK) | Cell Signaling Technology | 1:200 | 24991 | IHC |
| Phospho-STING (Ser366) | Cell Signaling Technology | 1:50 | 41622 | FC |
| Phospho-IRF3 (Ser386) | Cell Signaling Technology | 1:50 | 96421 | FC |

**Supplementary Table 6. List of antibodies**
