# Supplementary material for: Cell cycle inhibitors activate the hypoxia-induced DDX41/STING pathway to mediate antitumor immune response in liver cancer
Source: JCI Insight. 2024 Nov 22;9(22):e170532. doi: 10.1172/jci.insight.170532 (PMC11601891; doi:10.1172/jci.insight.170532)

Full unedited blot image for Figure 1E

γ-H2A.X (Ser139) antibody:

Ctrl Pac GSK AZD Palbo

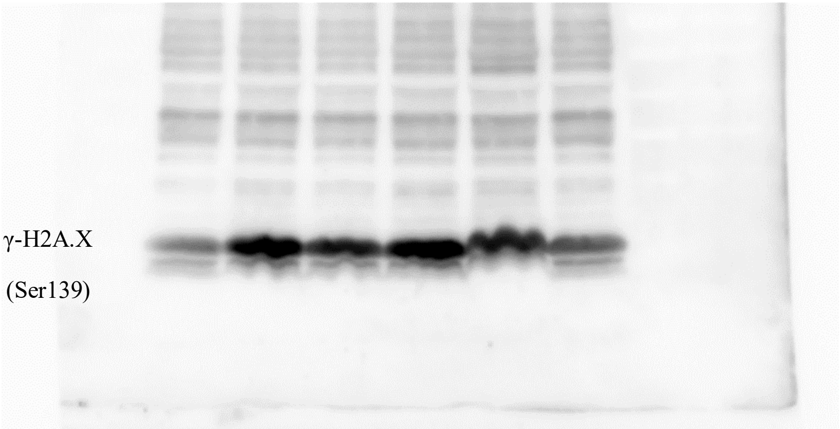

Pan-H3 antibody:

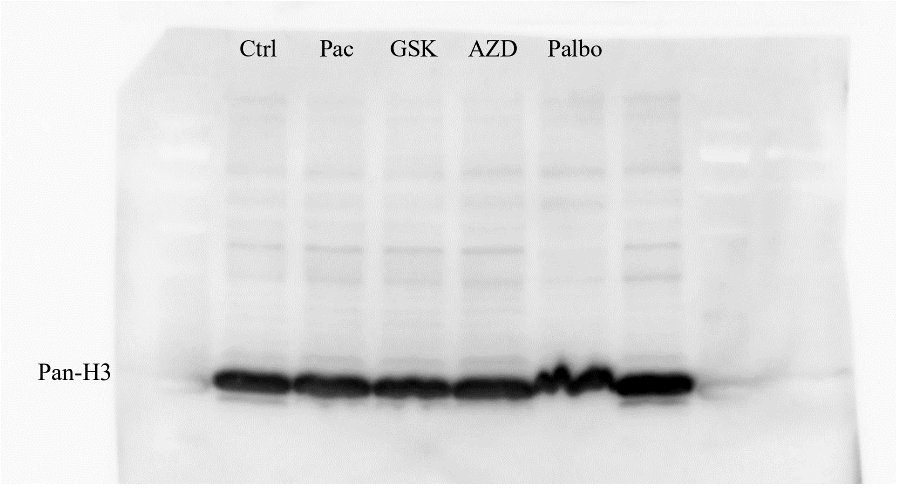

Full unedited gel for Figure 3C

For Paclitaxel panel:

STING antibody (STING dimer):

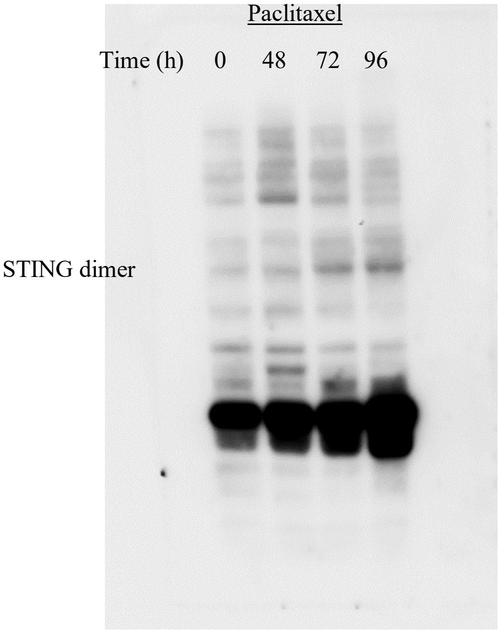

STING antibody (STING monomer):

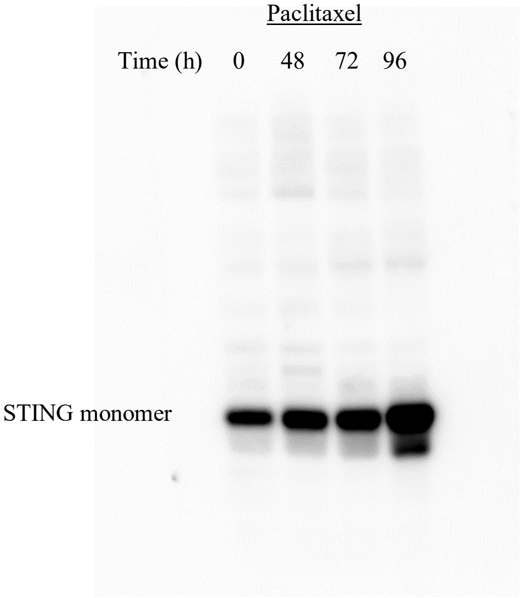

β-actin antibody:

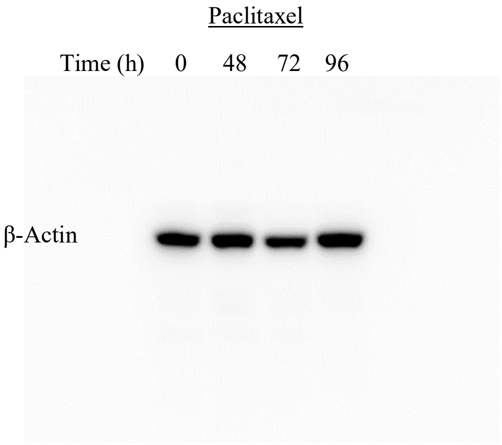

Full unedited gel for Figure 3C

For GSK1070916 panel:

STING antibody (STING dimer):

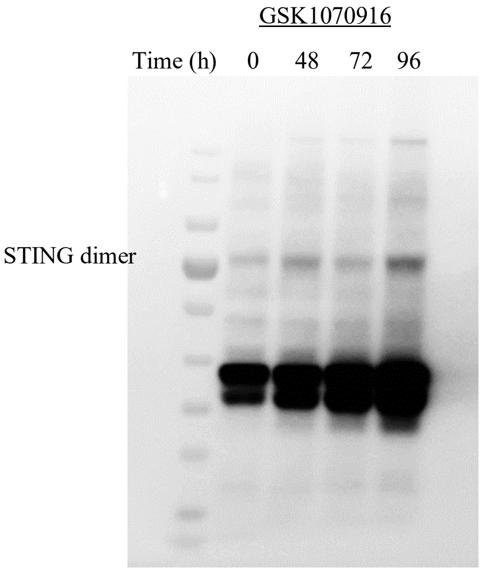

STING antibody (STING monomer):

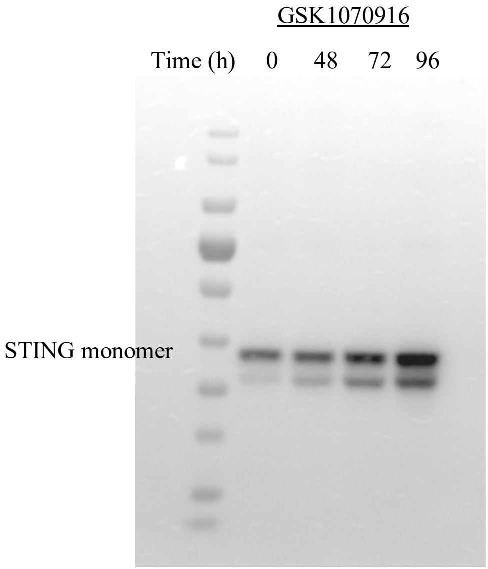

β-actin antibody:

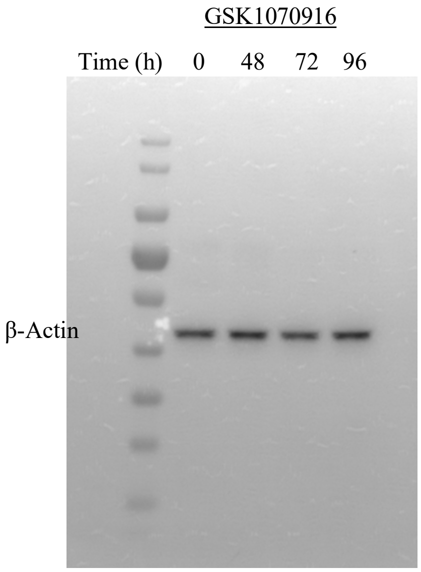

Full unedited gel for Figure 3C

For AZD1152 panel:

STING antibody (STING dimer):

AZD1152

Time (h) 0 48 72 96

STING dimer

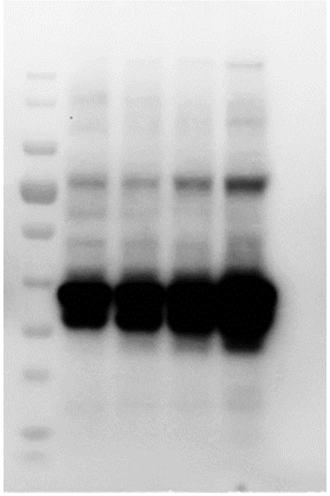

STING antibody (STING monomer):

AZD1152

Time (h) 0 48 72 96

STING monomer

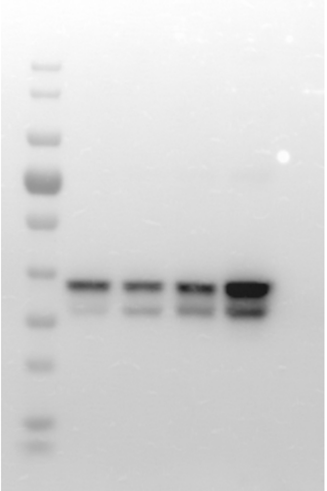

β-actin antibody:

AZD1152

Time (h) 0 48 72 96

β-Actin

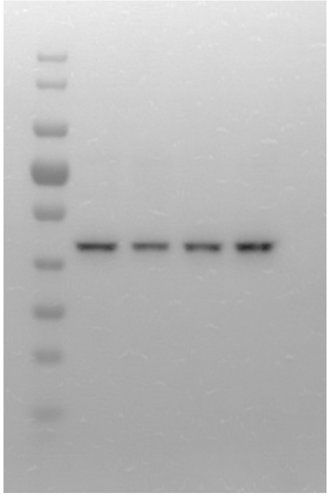

Full unedited gel for Figure 3C

For Palbociclib panel:

STING antibody (STING dimer):

Palbociclib

Time (h) 0 48 72 96

STING dimer

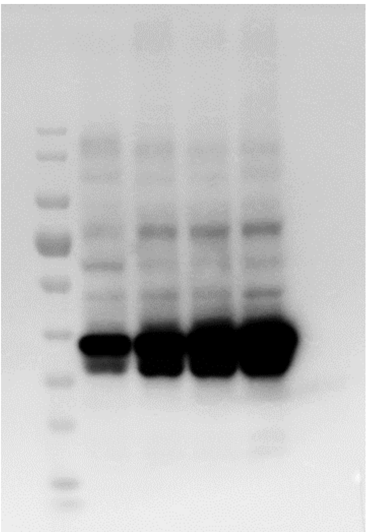

STING antibody (STING monomer):

Palbociclib

Time (h) 0 48 72 96

STING monomer

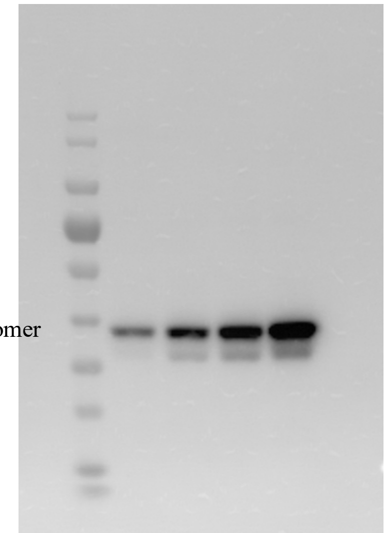

β-actin antibody:

Palbociclib

Time (h) 0 48 72 96

β-Actin

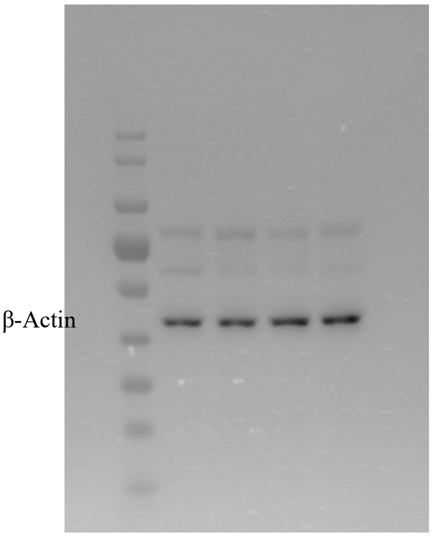

Full unedited gel for Figure 4G

For *DDX41* KD panel:

DDX41 antibody:

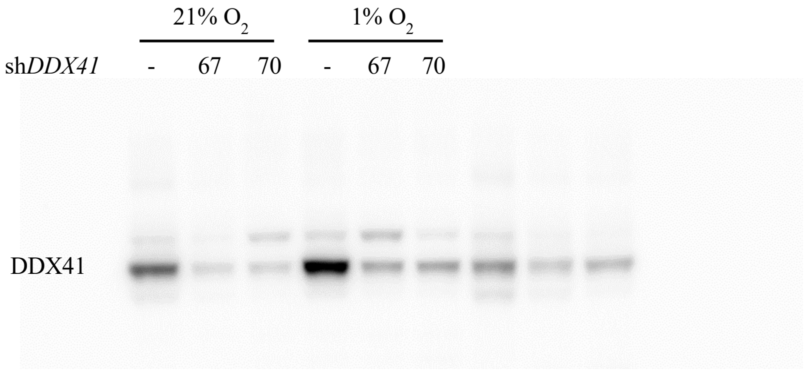

HIF-1α antibody:

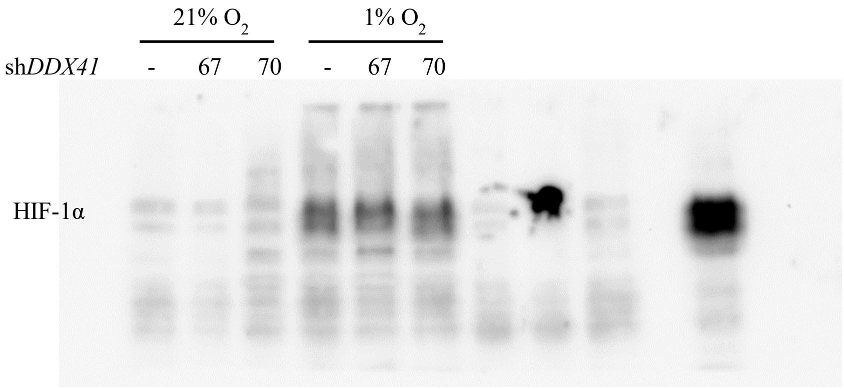

β-actin antibody:

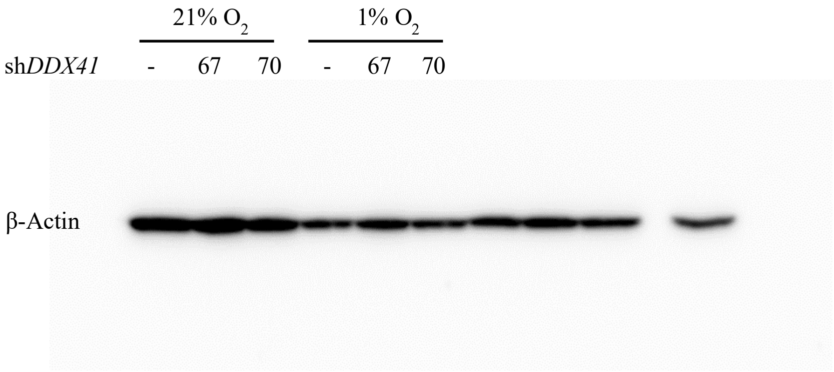

Full unedited gel for Figure 4G

For *HIF1A* KO panel:

DDX41 antibody:

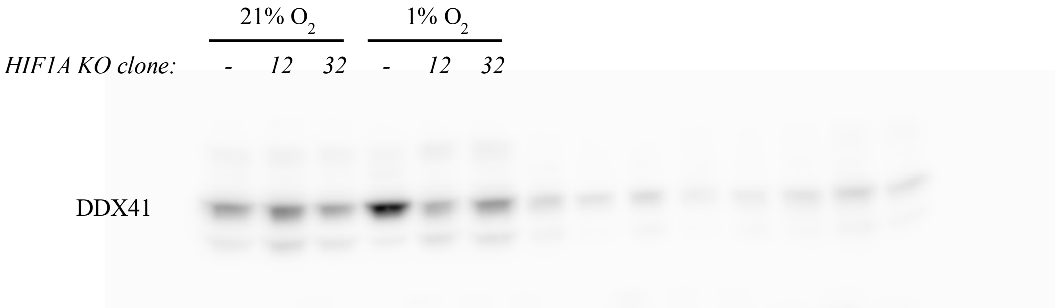

HIF-1α antibody:

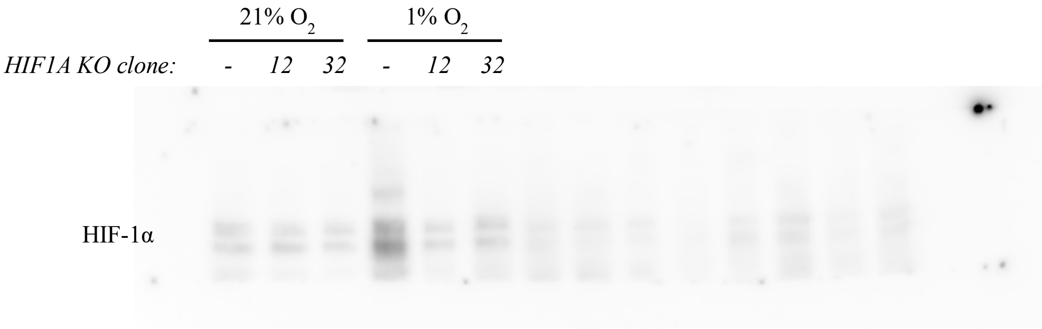

β-actin antibody:

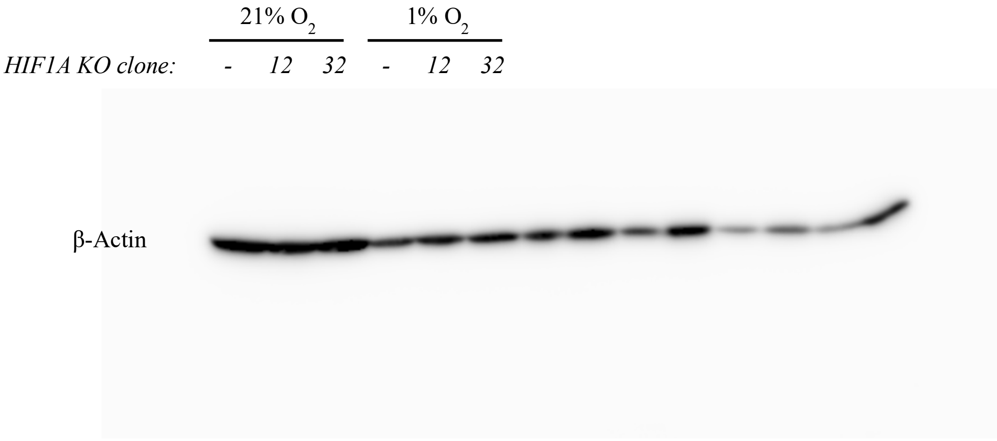

Supplement: Unedited blot and gel images [file jciinsight-9-170532-s307.pdf]
